# TAL Effector Specificity for base 0 of the DNA Target Is Altered in a Complex, Effector- and Assay-Dependent Manner by Substitutions for the Tryptophan in Cryptic Repeat –1

**DOI:** 10.1371/journal.pone.0082120

**Published:** 2013-12-03

**Authors:** Erin L. Doyle, Aaron W. Hummel, Zachary L. Demorest, Colby G. Starker, Daniel F. Voytas, Philip Bradley, Adam J. Bogdanove

**Affiliations:** 1 Department of Plant Pathology and Microbiology, Iowa State University, Ames, Iowa, United States of America; 2 Department of Genetics, Cell Biology & Development and Center for Genome Engineering, University of Minnesota, Minneapolis, Minnesota, United States of America; 3 Cellectis Plant Sciences, New Brighton, Minnesota, United States of America; 4 Division of Public Health Sciences, Fred Hutchinson Cancer Research Center, Seattle, Washington, United States of America; 5 Plant Pathology and Plant-Microbe Biology, Cornell University, Ithaca, New York, United States of America; Universität Stuttgart, Germany

## Abstract

TAL effectors are re-targetable transcription factors used for tailored gene regulation and, as TAL effector-nuclease fusions (TALENs), for genome engineering. Their hallmark feature is a customizable central string of polymorphic amino acid repeats that interact one-to-one with individual DNA bases to specify the target. Sequences targeted by TAL effector repeats in nature are nearly all directly preceded by a thymine (T) that is required for maximal activity, and target sites for custom TAL effector constructs have typically been selected with this constraint. Multiple crystal structures suggest that this requirement for T at base 0 is encoded by a tryptophan residue (W232) in a cryptic repeat N-terminal to the central repeats that exhibits energetically favorable van der Waals contacts with the T. We generated variants based on TAL effector PthXo1 with all single amino acid substitutions for W232. In a transcriptional activation assay, many substitutions altered or relaxed the specificity for T and a few were as active as wild type. Some showed higher activity. However, when replicated in a different TAL effector, the effects of the substitutions differed. Further, the effects differed when tested in the context of a TALEN in a DNA cleavage assay, and in a TAL effector-DNA binding assay. Substitution of the N-terminal region of the PthXo1 construct with that of one of the TAL effector-like proteins of *Ralstonia solanacearum*, which have arginine in place of the tryptophan, resulted in specificity for guanine as the 5’ base but low activity, and several substitutions for the arginine, including tryptophan, destroyed activity altogether. Thus, the effects on specificity and activity generated by substitutions at the W232 (or equivalent) position are complex and context dependent. Generating TAL effector scaffolds with high activity that robustly accommodate sites without a T at position 0 may require larger scale re-engineering.

## Introduction

Transcription activator-like (TAL) effectors (reviewed in 1) are a class of DNA binding transcription factors injected into host cells by members of the plant pathogenic bacterial genus *Xanthomonas* for targeted activation of specific host genes during infection. They are also important tools for biological engineering and genome editing because they can be readily engineered to bind specifically to almost any DNA sequence of interest. TAL effector binding site specificity is determined by a central repeat region (CRR) composed of a variable number of tandem repeats each typically 33-34 amino acids in length. The repeats are nearly identical, with repeat-to-repeat variation occurring primarily at amino acids 12 and 13 (termed the repeat-variable diresidue or RVD). The sequence of RVDs corresponds one-to-one with the sequence of bases on one strand of the target DNA, with the second residue of each RVD specifying a particular base through specific contacts or steric preclusion of alternate bases [2-5]. The bases of the DNA target that are encoded by the RVDs are known as the effector binding element, or EBE. Discovery of the TAL effector-DNA binding code has enabled the identification of candidate EBEs for specific TAL effectors, which facilitates the identification of TAL effector-targeted plant genes that play important roles in diseases caused by *Xanthomonas* *spp*. And the repeat-encoded targeting mechanism has proven to be modular, with no qualitative neighbor or context effects on specificity, making it possible to generate custom TAL effectors with desired specificities by assembling a CRR with an RVD sequence that corresponds to the intended target site (e.g. 6). 

Custom TAL effectors as transcription factors have been used to activate transcription of specific genes in plant and animal cells, using either the native TAL effector activation domain or replacing it with the VP16 activation domain from herpes simplex virus or its tetrameric derivative VP64 [7-10]. Targeted gene repression using custom TAL effectors has also been demonstrated, either by simply removing the activation domain or replacing it with a repressor domain [11-13]. TAL effectors fused to the catalytic domain of the endonuclease *Fok*I have been used extensively for genome editing. These TAL effector-nucleases (TALENs) [14], which function as paired monomers facing one another on opposite strands of the DNA, create double strand breaks (DSBs) precisely targeted to a short spacer between the two TAL effector binding sites [7,14-16]. In eukaryotic cells, such DSBs are subsequently repaired by either non-homologous end joining (NHEJ), which often creates short indels and can be used for targeted gene knockouts, or by homology directed repair (HDR), which can be used to make specific sequence changes or insert new DNA [17-19]. TALEN-mediated NHEJ and HDR have been demonstrated in a wide variety of cell types and organisms (reviewed in 1,20). 

Despite the broad utility of custom TAL effectors, however, TAL effector targeting appears to be constrained by a general requirement for the EBE to be directly preceded (5’) by a thymine (T). The T at this “0^th^” position (T_0_) is found in all but one of the known TAL effector binding sites in nature [2,3], and in the few studied cases, replacing it with another base has been shown to dramatically reduce or eliminate TAL effector-driven gene activation as well as DNA binding [3,21,22]. The single known natural TAL effector EBE without a T at base 0, in the promoter of the rice gene *OsSWEET14* and targeted by TalC of *X. oryzae*, has a cytosine instead, and although the effect of substituting a T has not been tested directly, a perfect match EBE for TalC, with a T at base 0 and corrected mismatches at two other locations indeed showed higher activity [23]. Similarly, functional TALENs targeting sites with nucleotides other than T at position 0 have been reported, but their activity was not compared to equivalent targets with a T [7,24,25]. Due to the apparent requirement of the 0^th^ position T for maximal TAL effector activity and binding, target sites for custom TAL effectors and TALENs have typically been selected with this constraint.

**Figure 1:**
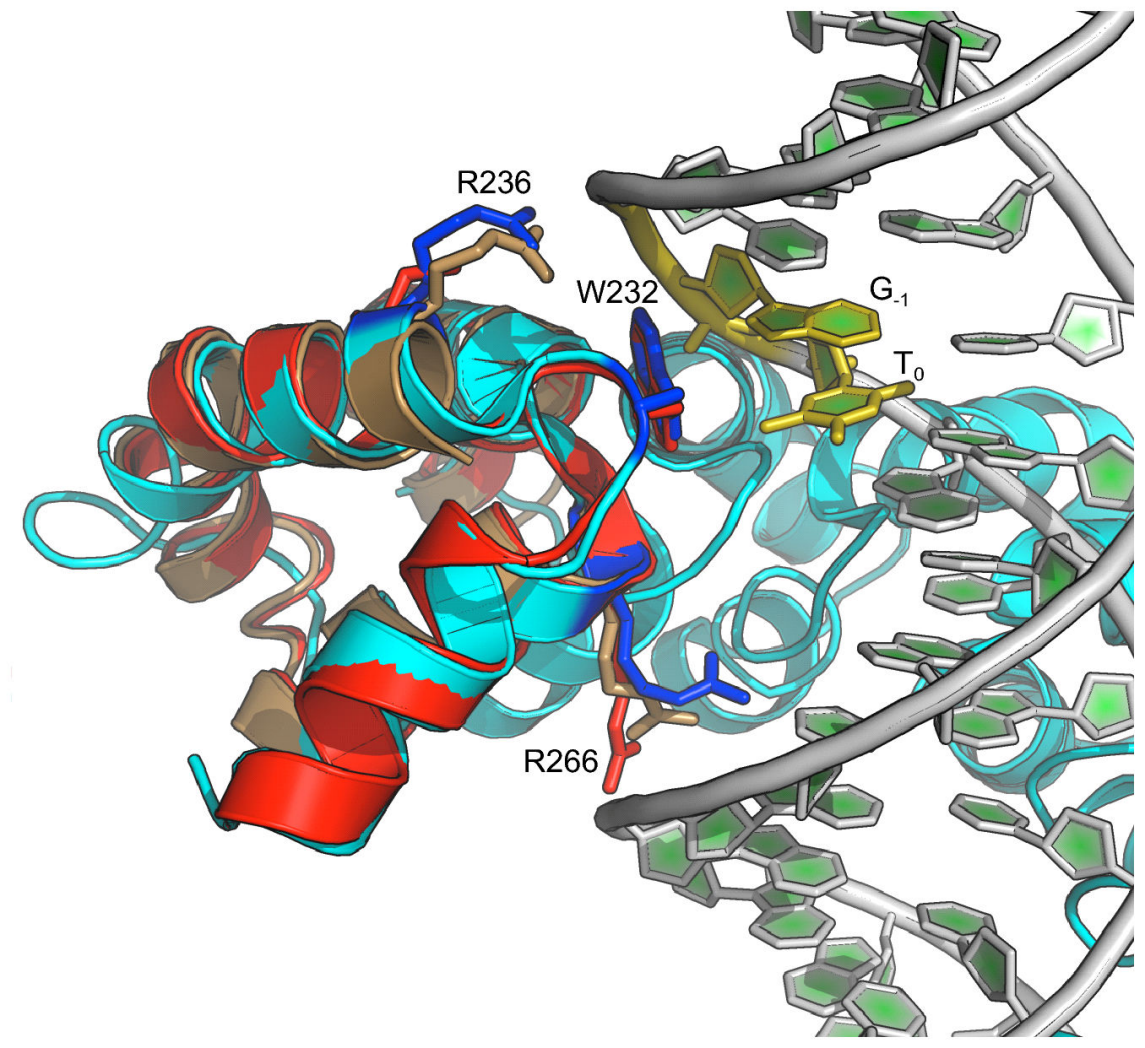
Alignment of N-terminal portions of multiple TAL effector crystal structures shows a conserved conformation for W232 consistent with an important interaction with the 0^th^ position T. The N terminus of TAL effector PthXo1 bound to its DNA target (PDB structure 3UGM) [4] is shown in blue, the N terminus of unbound artificial TAL effector dTALE2 (PDB structure 4HPZ) [26] in red, and the N terminus of artificial TAL effector dHAX3 bound to a DNA-RNA hybrid (PDB structure 4GG4) [5] in brown. DNA is from structure 3UGM. Side chains are shown for W232 as well as arginines at positions 236 and 266, which make non-specific contacts to the nucleic acid backbone. T_0_, the 0^th^ position thymine. G_-1_, a guanine 5’ of T_0_.

An explanation for the requirement for the 0^th^ position T was suggested by the crystal structure of *X. oryzae* TAL effector PthXo1 bound to its DNA target. This structure included two previously unrecognized cryptic repeats (designated the 0^th^ and -1^st^ repeats) located immediately N-terminal to the CRR that are similar in structure but dissimilar in sequence to the canonical repeats that encode the EBE. A tryptophan located in the -1^st^ repeat (W232), was revealed to be in close proximity to T_0_ and to form energetically favorable, base-specific van der Waals contacts with the methyl group of the base [4]. Although no other well-ordered, high resolution structures for the -1^st^ repeat region of a TAL effector bound to its DNA target have been reported, the role of W232 in specifying the 0^th^ position T is further supported by the structure of artificial TAL effector dHAX3 bound to a DNA-RNA hybrid [5], and by the unbound structure of artificial TAL effector dTALE2 [26], both of which show virtually no structural deviation from PthXo1 in the vicinity of W232 (Figure 1). Additionally, W232 and its surrounding residues are highly conserved in *Xanthomonas* TAL effector sequences, consistent with W232 playing an important role in TAL effector-DNA binding by encoding the conserved T at base 0 of natural EBEs. Truncation studies found that the portion of the TAL effector comprising repeats -1 and 0 is necessary for activity of both TAL effectors and TALENs [8,14,27], suggesting that these cryptic repeats are critical for TAL effector-DNA binding and further substantiating the hypothesis that specific engagement of T_0_ by W232 is important.

Here we report on a series of experiments to test directly whether the requirement for the 0^th^ position T depends on W232, and whether modifications of this position could enhance TAL effector-DNA targeting capacity by relaxing this requirement. Because the importance of the cryptic repeats in the N terminus prevents simply removing this portion of the protein to create TAL effectors (or TALENs) that have no requirement for T at the 0^th^ position, we sought to identify single amino acid substitutions for W232 that would relax or alter specificity for that base while retaining activity similar to wild type. For additional insight into base 0 specificity, we also examined the behavior of a chimeric protein consisting of a TAL effector CRR and C-terminus joined to the N-terminus of a *Ralstonia* TAL-like effector (RTL). RTLs, from the soil-borne, plant pathogenic bacterium *Ralstonia solanacearum*, are homologous with and functionally similar to TAL effectors, able to activate gene transcription and possessing CRRs composed of 35 amino acid repeats that, though divergent in overall sequence from TAL effector repeats [28,29], similarly mediate specific DNA recognition through RVDs [30]. Despite these marked similarities to TAL effectors, however, RTLs show sequence divergence throughout, and prefer targets with G at position 0 [30]. Our study reveals that W232 indeed specifies T_0_, but that effects of substitutions for W232 on base 0 specificity are influenced in a complex way by the composition of the central repeats, the N-terminal context, and whether TAL effector or TALEN activity or DNA binding is measured. We conclude that development of TAL effectors and TAL effector-based proteins with relaxed or altered specificities for enhanced targeting capacity will require larger scale engineering of the cryptic repeats, informed by a better understanding of intramolecular influences of the CRR and potentially other regions.

## Materials and Methods

### Construction of TAL effector W232 substitution variants

To generate PthXo1 TAL effector variants for *Agrobacterium*-mediated GUS reporter assays of activity, first the *Sph*I fragment containing the repeat region of the gene encoding TAL effector PthXo1 (clone obtained from B. Yang, Iowa State University) was ligated into the *Sph*I site of plasmid pCS466. pCS466 is a Gateway entry plasmid containing the *X. oryzae* pv. oryzicola *tal1c* gene, missing the *Sph*I fragment that contains its CRR [31]. The resulting plasmid, pAH103, therefore encodes a full length TAL effector with the PthXo1 CRR. To make single amino acid substitutions for W232, the region of pAH103 surrounding W232 was amplified via PCR using forward primer P885 (5'-GCGTCGGCAAACAGGCGTCCGGCGCACGC-3') crossing a *Not*I site, and mutagenic reverse primers crossing a *Stu*I site and each introducing a substitution at the codon for W232 and a silent *Xho*I site to facilitate screening (5’-CGTGAGCAAGGCCTCCAGGGCTCGAGCGCCGGANNNCTGTTTGCCG-3’; Ns represent the codon at position 232, detailed in Table S1). PCR products carrying the W232 substitutions were digested with *Not*I and *Stu*I and swapped back into the pAH103 backbone between those sites.

**Figure 2:**
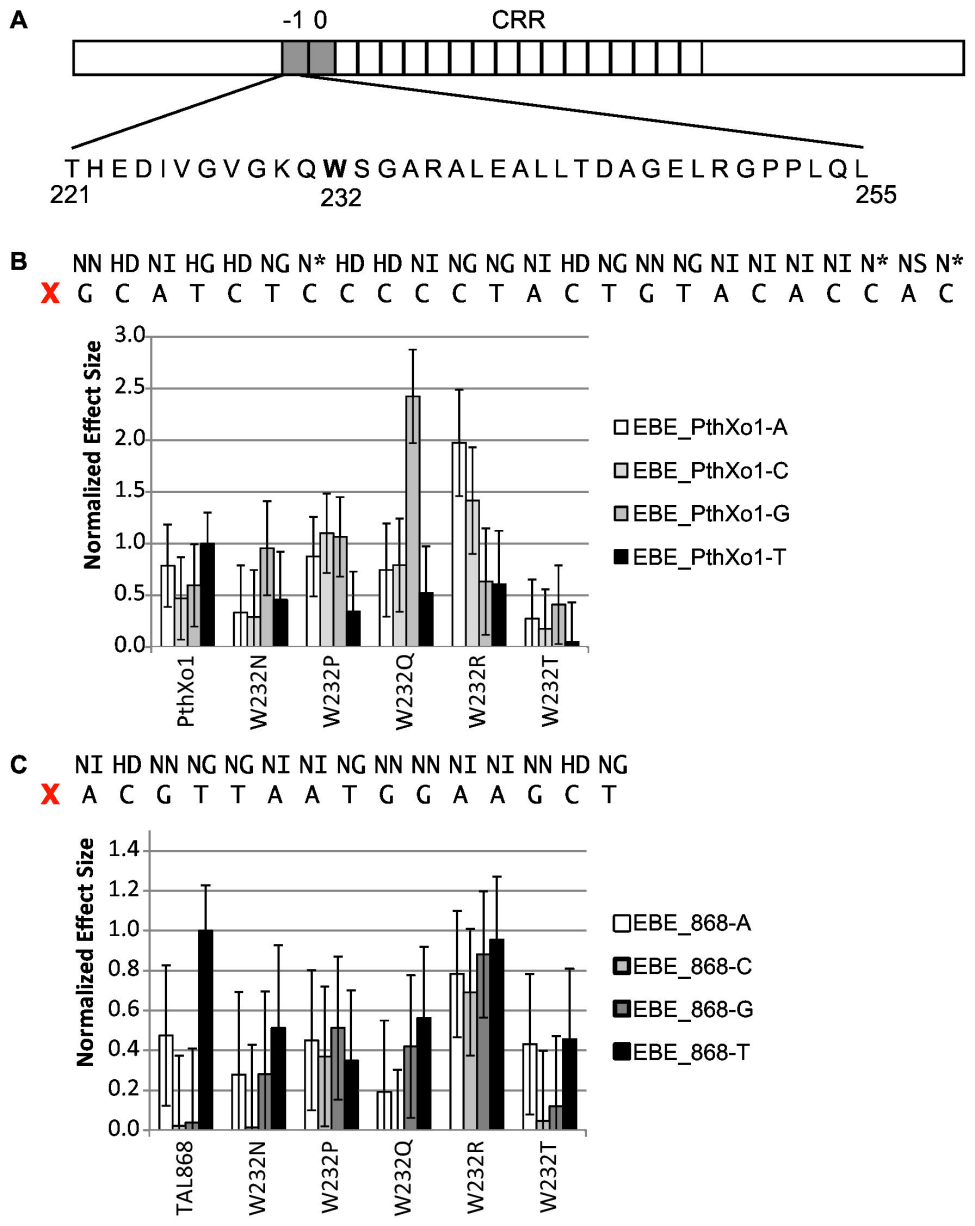
Activity of TAL effectors with selected single amino acid substitutions for W232 on targets with A, C, G, or T at the 0^th^ position. A. Schematic of a TAL effector with the -1^st^ and 0^th^ repeats and the central repeat region (CRR) labeled. The amino acid sequence of the -1^st^ repeat is shown below; W232 is shown in bold. B. Effects of PthXo1 W232 substitution and target combinations (treatment) on activity. Shown at top are the PthXo1 RVD and EBE sequences. X marks the 0^th^ position. Activity was measured in an *Agrobacterium*-mediated transient expression assay in *Nicotiana benthamiana* leaves, using a GUS reporter gene cloned downstream of a minimal promoter (see Materials and Methods) containing a PthXo1 EBE with the 0^th^ position thymine (EBE_PthXo1-T), or variants with adenine, cytosine, or guanine as base 0 (EBE_PthXo1-A, EBE_PthXo1-C, and EBE_PthXo1-G, and respectively). Treatment effects were estimated using a mixed linear model to account for variation due to experiment and replicate effects (see Materials and Methods). Effects were computed relative to the negative control EBE_PthXo1-T with no TAL effector and normalized to the effect of wild-type PthXo1 (W232) on EBE_PthXo1-T. Bars indicate one standard deviation. C. Effects of TAL868 W232 substitution and target combinations on activity, as in B. Shown at top are the TAL868 RVD and EBE sequences. X marks the 0^th^ position.

To generate TAL868 variants, a TALEN construct containing the TAL868 repeat sequence (Figure 2B) that was constructed using our Golden Gate method [32] was digested with restriction enzymes *Stu*I and *Aat*II to release a 1.7 kb fragment containing the repeat region. This fragment was then used to replace the PthXo1 CRR in pAH103 W232 substitution variants cut with the same enzymes. For *Agrobacterium*-mediated transient expression, the PthXo1 and TAL868 constructs were moved into the binary vector pGWB5 [33] using Gateway LR Clonase II (Life Technologies) according to manufacturer instructions.

### Construction of chimeric TAL effector RTL-PthXo1

DNA encoding a *Ralstonia* TAL-like effector N-terminal sequence was PCR-amplified with Phusion DNA polymerase (Thermo Fisher Scientific) from *R. solanacearum* GMI1000 genomic DNA (gift of D. Gross, University of Georgia) with forward primer P1178 (5’-TTGCATGTAAATAGGAGGTGCACCATGAGAATAGGCAAATCAAG-3’), which added sequence upstream of the start codon to match that of the *Xanthomonas* TAL effector expression constructs, and P1179 (5’-GAGACTCGTCTCGGCACGCGTGAGCTTCC-3’), which added a downstream *Esp*3I restriction site for cloning. This amplicon was tailed with 3’ adenine at each end using Taq Polymerase (Thermo Fisher Scientific) and cloned into the pCR8/GW/TOPO TA vector (Life Technologies). The cloned sequence was then released with *Esp*3I and *Eco*RV and substituted for the *Ban*I-*Eco*RV fragment of pAH103 to produce a full-length, Gateway-ready *Ralstonia*-*Xanthomonas* chimeric TAL effector construct in the Gateway entry vector, designated pAH410. Amino acid substitution variants of the chimeric effector construct were generated similarly to those for PthXo1 by PCR-amplifying a portion of the *Ralstonia* sequence using forward primer P1178 and a different mutagenic primer for each of five different amino acid substitutions at R298 (5’- CGGGTAGCAGCGCTTGCAGCGCCAGGTCACCCGANNNCTGC-3’, where Ns represent the codon for the amino acid at 298; full list in Table S2). PCR amplifications were done using Phusion DNA polymerase (New England Biolabs, Inc.). Each amplicon was digested with *Afe*I and *Bsa*I and swapped back in between those sites in pAH410. The chimeric effector and amino acid substitution variants were transferred to the binary expression vector pGWB5 [33] via the Gateway LR II Clonase enzyme kit (Life Technologies) for use in GUS assays.

### GUS assay of TAL effector activity

#### Construction of GUS reporter plasmids

For GUS reporter constructs, a 343 bp fragment of the *Bs3* promoter [21] was PCR amplified and ligated into the Gateway vector pCR8/GW/TOPO TA (Life Technologies), and site-directed mutagenesis was used to introduce an *Asc*I site upstream of the naturally occurring binding site for TAL effector AvrBs3. This modified promoter was recombined into the Gateway GUS reporter vector pGWB3 [33] upstream of the GUS gene using LR Clonase II (Life Technologies). Single-stranded DNA oligonucleotides containing the naturally occurring effector binding element (EBE) for PthXo1 (EBE_PthXo1) [2] or the TAL868 EBE (EBE_868) with A, G, C, or T at the 0^th^ position and with *Asc*I-compatible ends (Table S3) were annealed and ligated into the *Asc*I site.

#### Quantification of GUS activity

To measure TAL effector activity, TAL effector and reporter constructs were transformed into *A. tumefaciens* GV3101. Cells were grown overnight and diluted to OD_600_ = 0.8 in infiltration buffer (10 mM MgCl_2_, 5 mM MES pH 5.3, 200 µM acetosyringone). Cells carrying TAL effector constructs and cells carrying GUS reporter constructs were mixed 1:1 and infiltrated into the leaves of 6-8 week old *Nicotiana benthamiana* plants using a needleless syringe, and infiltrated areas were marked. After 48 hours GUS measurements were carried out based on a previously described protocol [34]. GUS activity was recorded as specific activity, calculated in relation to total protein, which was measured by Bradford assay (Bio-Rad).

#### Experimental design and initial screening of PthXo1 W232 substitution variants

During a single GUS experiment, TAL effector and reporter constructs were co-infiltrated into leaves on each of five different *N. benthamiana* plants for a total of five different measurements (leaf disks) per TAL effector-target combination per experiment. Each experiment included one to three TAL effector constructs co-delivered individually with each of the four corresponding target reporter constructs (differing at the position 0 base). In addition, all experiments included the target reporter construct with T at base 0 infiltrated alone as a negative control, and that reporter co-delivered with the corresponding wild-type (W232) TAL effector construct as a positive control.

For initial screening, each of the 19 PthXo1 W232 substitution constructs was tested against all four EBE_PthXo1 targets in at least one experiment. Within each experiment, GUS activity for each treatment (a TAL effector co-delivered with a target) was reported as the average of three leaf discs (high and low measurements were thrown out). To facilitate comparisons across experiments, which were conducted on different days and using different plants, data within each experiment were normalized to the positive control. 

Following the initial screening of PthXo1 mutants, the activity of five W232 substitution variants (selected based on the initial screening results) against EBE_PthXo1 with A, C, G, or T in position 0 was reassessed. Each variant was tested in at least three experiments, with different experiments carried out on different days. GUS assays were repeated in the same way for the five W232 substitution variants of TAL868. The additional PthXo1 experiments and the TAL868 experiments were carried out as described above; however, GUS activity measurements for all five leaf discs were retained and a mixed linear model analysis was carried out to discern significant effects (see below).

#### Mixed linear modeling of GUS activity

Because GUS assay results varied across replicates on different plants and across experiments, a mixed linear model was fit to the GUS activity data using the R package *lme4*. This model included fixed effects for treatment (each co-delivered TAL effector-target combination was considered to be one treatment) and random effects for experiment (day) and plant. GUS activity reported for individual leaf disks was treated as the response variable. All five leaf disk measurements for each treatment as well as the positive and negative controls from the corresponding experiments were included in the model (see Data File S1). Similar to a linear regression, fitting the mixed linear model allowed computation of a single estimate of the expected value of the effects of each treatment on GUS activity, independent of the effects of differing plants or experimental days. To identify significant differences between the effects of treatments on GUS activity, differences between the estimated treatment effects and *p*-values for those differences were computed using a general linear hypothesis (function *glht* from the R package *multcomp*) to test the null hypothesis that the effect of Treatment 1 on GUS activity is equal to the effect of Treatment 2 (see below and Script S1).

First, the difference in GUS activity between the negative control and each W232 substitution was estimated using contrast statements and the *glht* method to test the null hypothesis that activity of the appropriate negative control (either EBE_PthXo1-T or EBE_868-T with no TAL effector) was equal to the activity of a treatment. The test was performed for each treatment (a TAL effector W232 substitution variant co-delivered with a single target reporter construct) individually. The estimated differences between each treatment and the negative control and standard errors for those differences were then normalized to the estimated difference in effect size between the corresponding positive (EBE_PthXo1-T + PthXo1 W232 or EBE_868-T + TAL868 W232) and negative control for plotting.

To compare activity of the W232 substitutions on different targets with the activity of the (unsubstituted) type TAL effector on the target with T at position 0, the same mixed linear model and method was used to test for differences between the effect of each W232 TAL effector variant on each target (A, C, G, or T at base 0) individually and the corresponding wild-type TAL effector on the target with T at position 0 (i.e., four tests per W232 substitution variant). For each individual treatment, the null hypothesis tested stated that effect of the treatment on GUS activity was equal to the effect on activity of the corresponding wild-type TAL effector co-delivered with the target with T in the 0th position. 

Finally, the W232 substitutions were tested for altered or relaxed specificity for the 0^th^ position T. For each W232 substitution variant, the null hypothesis was that the effect on GUS activity of the substitution variant co-delivered with the target with A, C, or G in the 0^th^ position was equal to the effect of the same W232 substitution on the target with T in the 0^th^ position (targets with A, C, and G at position 0 were tested individually, for a total of 3 tests per substitution variant). Estimated differences in effect sizes and *p*-values for all tests are reported in the Information.

### TALEN assays

**Figure 3:**
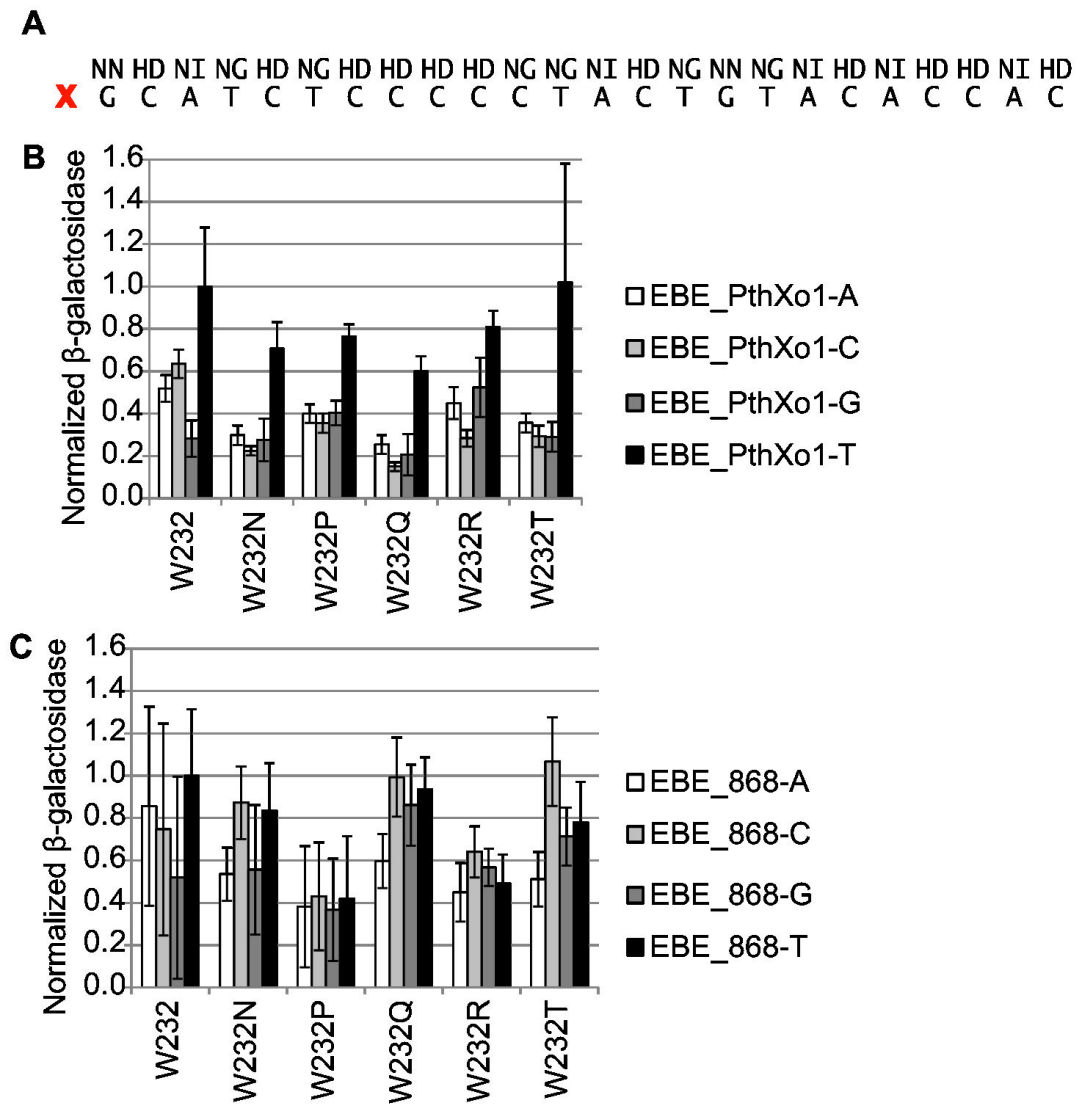
Activity of TALENs with selected single amino acid substitutions for W232 on targets with A, C, G, or T at the 0^th^ position. A. RVD sequence of the golden gate-assembled PthXo1 equivalent and nucleotide sequence of the PthXo1 EBE. X marks the 0^th^ position. B. Activity of TALENs with the PthXo1 equivalent RVD sequence, in a yeast single strand annealing assay [14]. EBE_PthXo1-A, EBE_PthXo1-C, EBE_PthXo1-G, and EBE_PthXo1-T indicate activity on targets with paired PthXo1 EBEs each with A, C, G, or T at the 0^th^ position, respectively. Data are normalized to PthXo1 on EBE_PthXo1-T. Values are the mean of seven or more replicates. Error bars represent s.d. See Table S8 for *p*-values.

 We used a previously described yeast-based single-strand annealing assay of TALEN activity [32,35] to test the effects of substitutions for W232 on PthXo1 and TAL868-based TALENs. Briefly, *a* mating-type cells containing TALEN expressing plasmid were mated to α mating-type cells containing target plasmids. Cleavage of the target plasmid results in reconstitution of a functional *lacZ* gene, providing a quantitative measurement of TALEN activity. All data were normalized to the wild-type (W232) TALEN on the target with 5’ T. Data represent the averages of at least five replicates. Experiments were repeated twice. TALEN constructs were made by assembling repeats for a PthXo1 equivalent (Figure 3A) or for TAL868 (Figure 2C) into pZHY500 [36] and each of five W232 substitution derivatives of that vector. The constructs were assembled by the Golden Gate method [6], using RVDs HD, NG, NI, and NN to exactly match the corresponding targets. The PthXo1 equivalent therefore varies slightly from the wild-type PthXo1, which contains some less common RVDs and some RVDs mismatched to the target. The pZHY500 derivatives were made by amplifying a fragment of the TAL effector backbone construct in that vector spanning the W232 codon, using forward primer 5’-GGACGCAAGTGGTTGGTCTAGAATGGTGG-3’ and each of five mutagenic reverse primers (5’-CCTCCAGGGCGCGTGCGCCGGANNNCTGTTTGCCGACGCC-3’, where Ns represent the codon corresponding to W232, detailed in Table S4). PCR products were digested with *Xba*I and *Stu*I and swapped in between those sites in pZHY500 (or pZHY501, which differs only by the yeast marker used) [36]. Target plasmids were constructed as previously described [6], using homodimeric target sites consisting of EBE_PthXo1 (Figure 3A) or EBE_868 (Figure 2C) with A, C, G, or T at position 0. 

### TAL effector DNA binding affinity assays

#### Expression and purification of recombinant TAL effector proteins

A bacterial expression vector pGEX6P2-TALE was created by ligating a Golden Gate compatible TAL effector backbone construct truncated 5’ at codon 152 and 3’ at codon 63 following the repeat region into pGEX6P2 (GE Healthcare). Variants with substitutions at W232 were created by site-directed mutagenesis (Quickchange II, Agilent) using the same method and primers described above for the TALEN yeast assays (Table S4). Repeats for PthXo1 and TAL868 were assembled into pGEX6P2-TALE and each of the substitution derivatives using the Golden Gate method as previously described [32]. These expression constructs were then transformed into Rosetta (DE3) pLysS cells (EMD Millipore) and selected on media containing carbenicillin (50 µg/ml) and chloramphenicol (30 µg/ml). 200 mL cultures were grown to log phase at 37°C before induction for 3 hours with 1 mM IPTG. The cells were pelleted by centrifugation and lysed in GST lysis buffer (25 mM HEPES pH 7.4, 150 mM NaCl, 5 mM MgCl_2_, 130 µM CaCl_2_, 0.5% Triton X-100, 10% glycerol, 1 mM PMSF, 1 µg/mL Leupeptin, 100 nM Aprotinin, 1 µg/mL Pepstatin A). The lysates were treated with RNase A (20 µg/mL) and DNase I (10 U/mL), clarified by centrifugation (21,000 x g, 10 minutes), and then loaded onto a column containing equilibrated Glutathione Sepharose (GE Healthcare). The columns were washed with GST lysis buffer and subsequently by cleavage buffer (50 mM Tris-HCl pH 8.0, 1 mM EDTA, 1 mM DTT, 10% glycerol). Elution of untagged purified TAL effector protein was performed by overnight incubation at 4°C with PreScission protease (GE Healthcare). Eluted TAL effector proteins, separated by electrophoresis and stained with Coomassie blue, were stable and uniformly >95% pure (Figure S1), so subsequent quantification was performed by Bradford assay (Bio-Rad). 

#### Electrophoretic mobility shift assay (EMSA)

Double stranded DNA substrates were prepared by annealing fluorescently tagged complementary oligonucleotides. Sequences for substrates used were 5′- tggacacgacttgagcNACGTTAATGGAAGCTcgtagtgctgtgctga-3’ with N=A for EBE_868-A, N=C for EBE-868-C, N=G for EBE_868-G, and N=T for EBE_868-T, or 5′- tggacacgacttgagcTCGACGCTCAGGCAACcgtagtgctgtgctga-3’ for the scrambled target. Purified proteins were diluted into binding buffer (10 mM HEPES pH 7.6, 10% glycerol, 100 mM KCl, 10 mM MgCl_2_, 100 µM EDTA, 500 µ M DTT, 15 ng/µL salmon sperm DNA, 30 ng/µL BSA) at varying concentrations (500, 250, 125, 62.5, 31.25, 15.625, 7.1825, and 0 nM), with a fixed concentration of the labeled DNA substrate (20 nM). The reactions were incubated for 30 minutes at room temperature and then separated by electrophoresis on a 7% TBE-acrylamide gel. Detection of the labeled substrate was then performed on a fluorescence scanner (Storm 860, Molecular Dynamics).

### Structural predictions

 DNA binding specificity calculations were performed for wild type and all 19 canonical amino acid variants using the molecular modeling package Rosetta [37]. The structure of PthXo1 bound to its natural target site (PDB ID: 3UGM) was used as a modeling template. Two levels of conformational sampling were explored, either keeping the protein backbone completely fixed or allowing limited flexibility in the neighborhood of W232. Each variant was simulated in complex with 16 different DNA target sites that sampled all possible DNA sequences at positions 0 (the canonical T position) and -1 (also contacted by W232 in the crystal structure). At the end of each simulation, a binding energy for the final structure was computed by taking the difference between the energy of the complex and the energies of the unbound partners, allowing limited conformational relaxation in the unbound state prior to computing the unbound energies.

## Results

### Single amino acid substitutions for W232 alter target specificity and activity of a TAL effector with the repeat region of PthXo1

To test whether W232 accounts for TAL effector specificity for T at the 0^th^ position of the binding site, we generated a full length TAL effector construct encoding the repeat region of *X. oryzae* TAL effector PthXo1 (Figure 2B) (see Materials and Methods; for simplicity hereafter referred to PthXo1) with the wild-type W232 as well as variants with all 19 possible single amino acid substitutions at this position. We then tested the ability of the PthXo1 W232 substitution variants, in an *Agrobacterium*-mediated transient expression assay in *N. benthamiana* leaves, to activate transcription of a GUS reporter gene cloned downstream of a minimal promoter (see Materials and Methods) containing the PthXo1 effector binding element (EBE) with the 0^th^ position thymine (EBE_PthXo1-T), or variants with adenine, cytosine, or guanine as base 0 (EBE_PthXo1-A, EBE_PthXo1-C, and EBE_PthXo1-G). In this and all other PthXo1 experiments presented in this article, the EBE used is the one found in the natural target of PthXo1, the rice *Os8N3* gene [38,39], which harbors 4 mismatches to the native PthXo1 RVD sequence (Figure 2B). 

In an initial screen, all 19 substitutions for W232 appeared to alter specificity for the 0^th^ position T, showing highest activity on a target with A, C, or G at that position instead of T, or showing nearly equal activity on all targets (Figure S2). Many of the substitutions reduced activity to less than half that of PthXo1 W232 on the target EBE_PthXo1-T. However, the arginine (W232R), asparagine (W232N), glutamine (W232Q), proline (W232P), and threonine (W232T) substitutions appeared to alter or relax specificity for T at the 0^th^ position while maintaining relatively high levels of activity. We selected these substitutions to characterize further.

### The effects of W232 substitutions on TAL effector activity and specificity at the 0^th^ position depend on the CRR

 To confirm the results of the initial screen and to next determine whether the effects of W232 substitutions on specificity and activity depend on the composition of the TAL effector CRR, we repeated the activation assays with the five selected PthXo1 substitution variants and made and tested equivalent constructs for a second TAL effector, TAL868, using variants of its (perfect match) EBE each with a different base at position 0 (EBE_868-A, EBE_868-C, EBE_868-G, and EBE_868-T) (Figure 2). The TAL868 CRR differs in both RVD sequence and repeat number from that of PthXo1. Assays were carried out with replicates within an experiment done on different plants and with independent experiments done on different days.

 Relative activity of the various TAL effector constructs on each of the corresponding promoters varied both within experiments (across plants used) and across experiments. To discern significant differences among the tested combinations, we constructed a mixed linear model for analysis (see Materials and Methods). The model used measured GUS activity as the response variable, and included fixed effects for treatment (a TAL effector and promoter combination) and random effects for plant and experiment. Similar to a linear regression, fitting this model allowed estimation of the effects of individual treatments (a co-delivered TAL effector-reporter combination was considered to be a treatment) on GUS activity independent of the effects of plant or experiment day. The model also allowed us to estimate differences in effects on GUS activity between treatments and to test for significant differences between treatments with a general linear hypothesis test (Materials and Methods).

Using this model, we estimated the effect on GUS activity of each W232 substitution co-delivered with each of the four targets, relative to the corresponding negative control, by estimating the difference between the effect sizes of the two treatments (Table S5). We then tested for differences between the estimated effects on GUS activity of each W232 substitution variant on each of the four targets and the wild-type TAL effector on the T target to examine activity in relation to “normal” (individual general linear hypothesis tests were carried out for the W232 substitution variant on each target; Table S6). Finally, we identified changes in 0^th^ position specificity by comparing activity of each TAL effector on the A, C, or G target to its activity on the T target (Table S7), and examining differences in the patterns of activity compared to that of the unsubstituted TAL effector. 

Both TAL effectors showed highest mean activity on their target with T at the 0^th^ position, though using a *p*-value cutoff of 0.05, this was significant only for TAL868 (Table S7). In general, W232 substitutions showed relaxed or altered specificity for base 0. However, changes to base 0 preference, or to activity, caused by a substitution differed between the two TAL effectors (Figure 2 and Tables S5-S7). 

For example (at *p*<0.05 unless otherwise noted), in the context of PthXo1 W232Q shifted specificity for base 0 to G (Table S7) and showed activity on that target nearly 2.5 times higher than activity of wild-type PthXo1 on EBE_PthXo1-T; Figure 2B and Table S6), while in the context of TAL868 it relaxed specificity entirely (activity on targets with 0^th^ position A, C, or G was not significantly different from activity on the target with T at position 0; Table S7) but reduced overall activity relative to TAL868 on EBE_868-T (the reduction was significant for the A and C targets; Table S6). W232R in PthXo1 showed a significant preference for the A target (p<0.05), with an apparent mean activity level nearly twice that of normal (*p*=0.582). In TAL868, however, this substitution relaxed specificity while maintaining near normal activity across all targets. Finally, W232N and W232T relaxed specificity in both TAL effectors (Table S7) and reduced activity, but the degree of reduction differed between the two TAL effector contexts (Figure 2 and Table S6). Since PthXo1 and TAL868 differ only in their repeats, altogether, the results indicate an effect of the TAL effector CRR (or the target EBE sequence, or both) on the behavior of variants with substitutions at W232 with regard to specificity and activity.

### Effects of W232 substitution on activity and specificity are different in TALENs compared to TAL effectors

 Because TALENs have rapidly become the most widely used application of custom TAL effectors, we next asked whether the observations we made regarding W232 substitutions in TAL effectors in the activation assay hold for TALENs in a yeast-based single-strand annealing assay of DNA cleavage activity [14]. In this assay the two monomers that make up a TALEN bind a pair of EBEs on opposing strands and separated by a short spacer, all between two partially duplicated terminal fragments of *lacZ*. This brings the nuclease domains together to cleave the spacer, stimulating recombinational restoration of *lacZ* by single strand annealing for a quantifiable readout (β-galactosidase activity) that serves as a proxy for TALEN DNA cleavage activity. We generated homomeric PthXo1- or TAL868-based TALEN pairs, and variants with each of the five substitutions tested above, and assayed their activity on targets with corresponding paired identical EBEs with A, C, G, or T at the 0^th^ position (Figure 3 and Table S8). The TALENs were assembled by our previously reported golden gate method using RVDs HD, NG, NI, and NN to match the corresponding EBE [6]. The CRR of the PthXo1 TALEN (Figure 3A) therefore differs slightly from the naturally occurring one used in the TAL effector assay described above, which in addition to the mismatches, contains some less common RVDs. The TAL effector domains in both TALENs (see Materials and Methods) correspond to the “Miller et al.” architecture [7], which, though truncated at both ends, contains the 0 and -1 repeats, as well as two additional cryptic repeats, -2 and -3, that complete the structurally well-ordered portion of the N-terminus that is involved in DNA binding [40]

 As expected, highest mean activity for both TALENs with W232 was on the targets with a T at the 0^th^ positions, though this preference was not statistically significant for TAL868 (Table S8). All of the W232 substitution variants for the PthXo1 TALEN also showed a preference for EBE_PthXo1-T, which was significant (*p*<0.05), but all but W232T were less active (Figure 3A). In the TAL868 context, the data were more variable, but each W232 substitution altered the pattern of activity on the different targets (Figure 3B). W232P in TAL868 showed no significant 0^th^ base preference and W232R a slight preference for A (*p*<0.05), but neither showed activity (on any target) greater than 60% of the activity of the W232 TALEN on EBE_868-T. W232N, W232Q, and W232T in TAL868 showed a different pattern, with highest mean activity of each being on the target preceded by C and lowest on A. The disfavor for A was significant for each variant but the preference for C was significant only for TAL868 W232T (p<0.05). The highest activity of each (on the target with C at position 0) was not significantly different from that of the unsubstituted TALEN on EBE_868-T. Overall then, the tested W232 substitutions in TALENs had either a negative or no affect on activity, and their effects on specificity were different from those observed in the activation assay. However, as in the activation assay, effects on both activity and specificity were influenced by the CRR.

### Substitutions for W232 in TAL868 reduce base 0 specificity and overall affinity in a DNA binding assay

**Figure 4:**
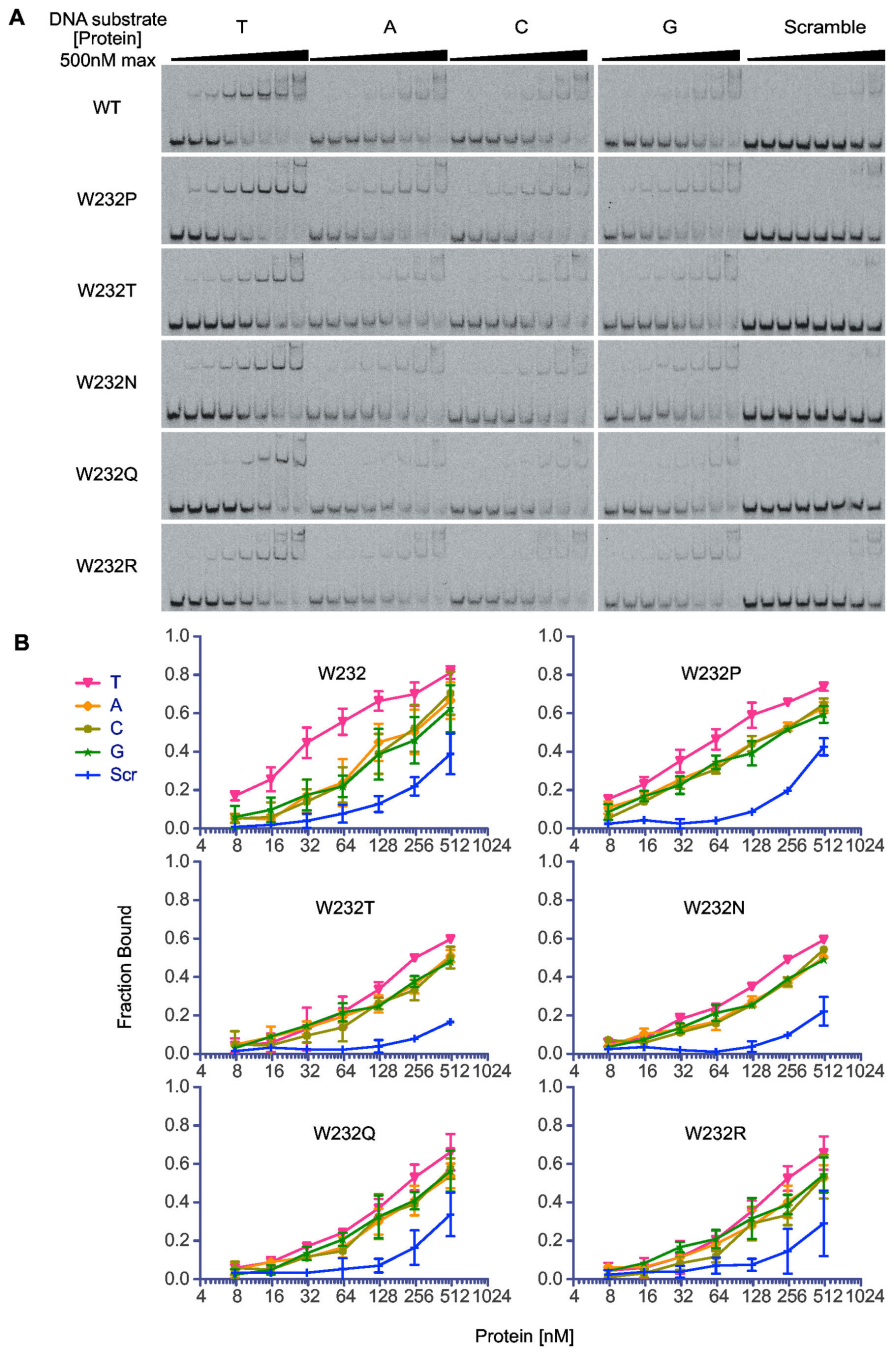
Relative binding affinities of TAL868 and selected W232 substitution variants for the TAL868 target DNA with T, A, C, or G at the 0^th^ position. A. Electrophoretic mobility shift assay of labeled TAL868 target DNA with T, A, C, or G in the 0^th^ position, or a scrambled target, (as labeled at top) following incubation with increasing amounts (left to right) of TAL868 (W232) or selected W232 substitution variants, as labeled. Bands across the bottom represent unbound DNA. The next bands up represent DNA bound by the TAL effector. The uppermost bands represent higher order complexes. DNA bound at lower protein concentrations indicates higher affinity. Each interaction was assayed at least twice. Representative gel images are shown. B. Results from all experiments, reported as the fraction of DNA bound by the protein, estimated by band densitometric analysis. Error bars represent standard deviation.

 Using an electrophoretic mobility shift assay (EMSA), we next tested whether the effects of W232 substitutions on TAL effector or TALEN activity and binding site specificity are directly related to changes in TAL effector-DNA binding affinity. We assembled the PthXo1 and TAL 868 CRRs into a tagged backbone construct for expression and purification that spans the portion of the TAL effector used in the TALEN constructs (see Materials and Methods). We were unable to obtain sufficient soluble PthXo1 protein for the EMSA, however TAL868 expressed well and was stable (Figure S1), so we made an additional TAL868 expression construct for each of the five substitution variants we had characterized in the TAL effector and TALEN activity assays. Each of these variant TAL868 proteins expressed similarly to the unsubstituted protein (Figure S1). The purified proteins were mixed individually with double stranded oligonucleotides comprising EBE_868-A, EBE_868-C, EBE_868-G, or EBE_868-T, over a range of protein concentrations, and the concentration at which the mobility of the DNA shifted was assessed as a measure of relative affinities (Figure 4). As expected, the TAL868 protein with the native W232 showed strongest relative affinity for EBE_868-T. Each W232 substitution variant showed a similar preference for T, but affinity of each for EBE_868-T was reduced relative to the W232 protein, and the preference for T was less pronounced. These changes in binding affinity and specificity do not match the changes in activity and specificity observed in the TAL effector and TALEN activity assays. Because we could not purify sufficient PthXo1, whether the effects of the substitutions on DNA binding are influenced by the composition of the CRR (or target) remains to be tested. 

### W232-equivalent substitutions in a chimeric RTL-TAL effector reveal an influence of N terminal sequence context on specificity for base 0

**Figure 5:**
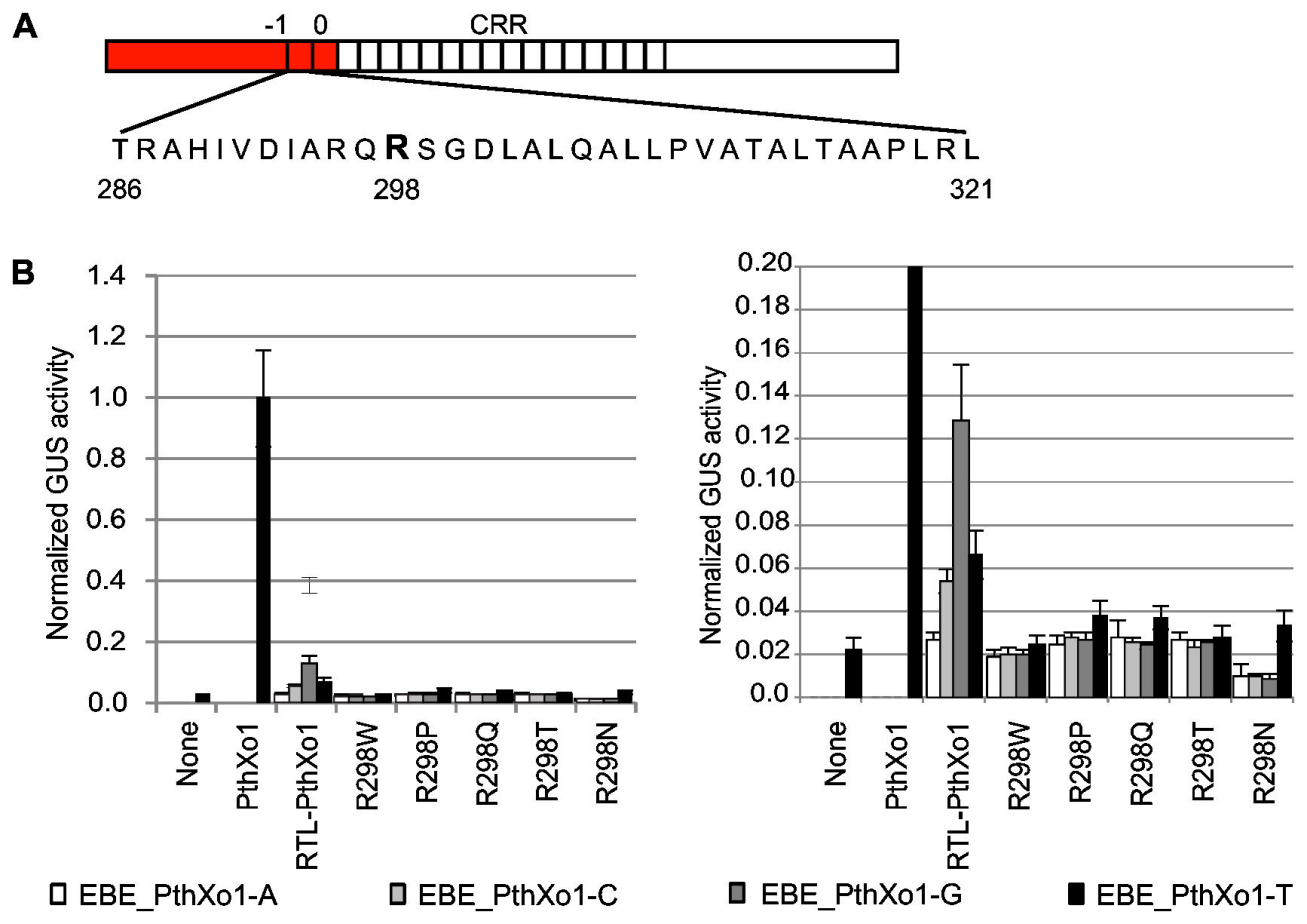
Activity of chimeric TAL effector RTL-PthXo1 with single amino acid substitutions for R298 on targets with A, C, G, or T at the 0^th^ position. A. Schematic of the chimeric TAL effector RTL-PthXo1 with the -1^st^ and 0^th^ repeats and the central repeat region (CRR) labeled, and showing the RSc1815 -1st repeat sequence. RTL-PthXo1 was created by replacing the N terminal region immediately upstream of the CRR of our PthXo1 construct with the N terminal region of RTL RSc1815 (shown in red). The arginine (R) in the RTL that aligns to W232 is shown in large bold font. Numbers indicate positions in the RTL sequence. B. Left, activity of RTL-PthXo1 with the native arginine at position 298 (R298) and of variants with selected amino acid substitutions at that position (as labeled), measured in an *Agrobacterium*-mediated transient expression assay in *Nicotiana benthamiana* leaves, using a GUS reporter gene cloned downstream of a minimal promoter (see Materials and Methods) containing the PthXo1 EBE with the 0^th^ position thymine (EBE_PthXo1-T), or variants with adenine, cytosine, or guanine as base 0 (EBE_PthXo1-A, EBE_PthXo1-C, and EBE_PthXo1-G, and respectively). Right, the same plot at a larger scale. Activity is normalized to the activity of PthXo1 on EBE_PthXo1-T, set to 1.0. Values are the mean of five replicates. Error bars represent s.d. See Table S9 for p-values. Experiments were repeated at least twice with similar results.

 Because our results in both the TAL effector and TALEN activity assays showed that base 0 specificity and activity of W232 substitution variants depend on the downstream CRR, we wanted to know whether N-terminal sequence differences surrounding W232 also could have an effect. To address this question, we chose to examine base 0 specificity of an RTL-TAL effector chimera created by replacing the N terminus of PthXo1 up to its (native, not Golden Gate-assembled) CRR with a corresponding fragment of RTL RSc1815 (GenBank ID CAD15517.1) [28] (Figure 5 and Table S9). Amino acid sequence alignment of three complete RTL sequences with PthXo1 (Figure S3) and predictions of secondary structures (Figure S4) indicated high similarity throughout the N terminal region. The residues immediately on either side of W232 in PthXo1 are conserved in the RTLs, but W232 itself aligns with an arginine (R298 in Rsc1815). The N termini each are predicted to form six helices, with four helices corresponding approximately to the -1^st^ and 0^th^ repeats of PthXo1. W232 in PthXo1 and the equivalent arginine in the RTLs are located on a short loop between the two helices that make up the -1^st^ repeat in each protein. In the GUS assay, the activity of the RTL-PthXo1 chimera was almost an order of magnitude lower than that of PthXo1, but the chimera showed a strong preference for G at the 0^th^ position (*p*<0.05). This preference agrees with a recent study of full length RTLs [30]. Notably, it contrasts with the result of W232R in PthXo1, which was preference for A. It also differs from the result of W232R in TAL868, which was a relaxation of specificity. We next assayed the effect of substituting tryptophan, asparagine, proline, glutamine, or threonine for R298 in the RTL-PthXo1 chimera. Each substitution, including the tryptophan, reduced activity to background levels. Thus, in addition to CRR composition, the N-terminal context influences the function of the W232 equivalent residue in determining TAL effector specificity for the 0^th^ position base.

### Structural models of W232 substitutions predict W232 and W232R as most energetically favorable

**Figure 6:**
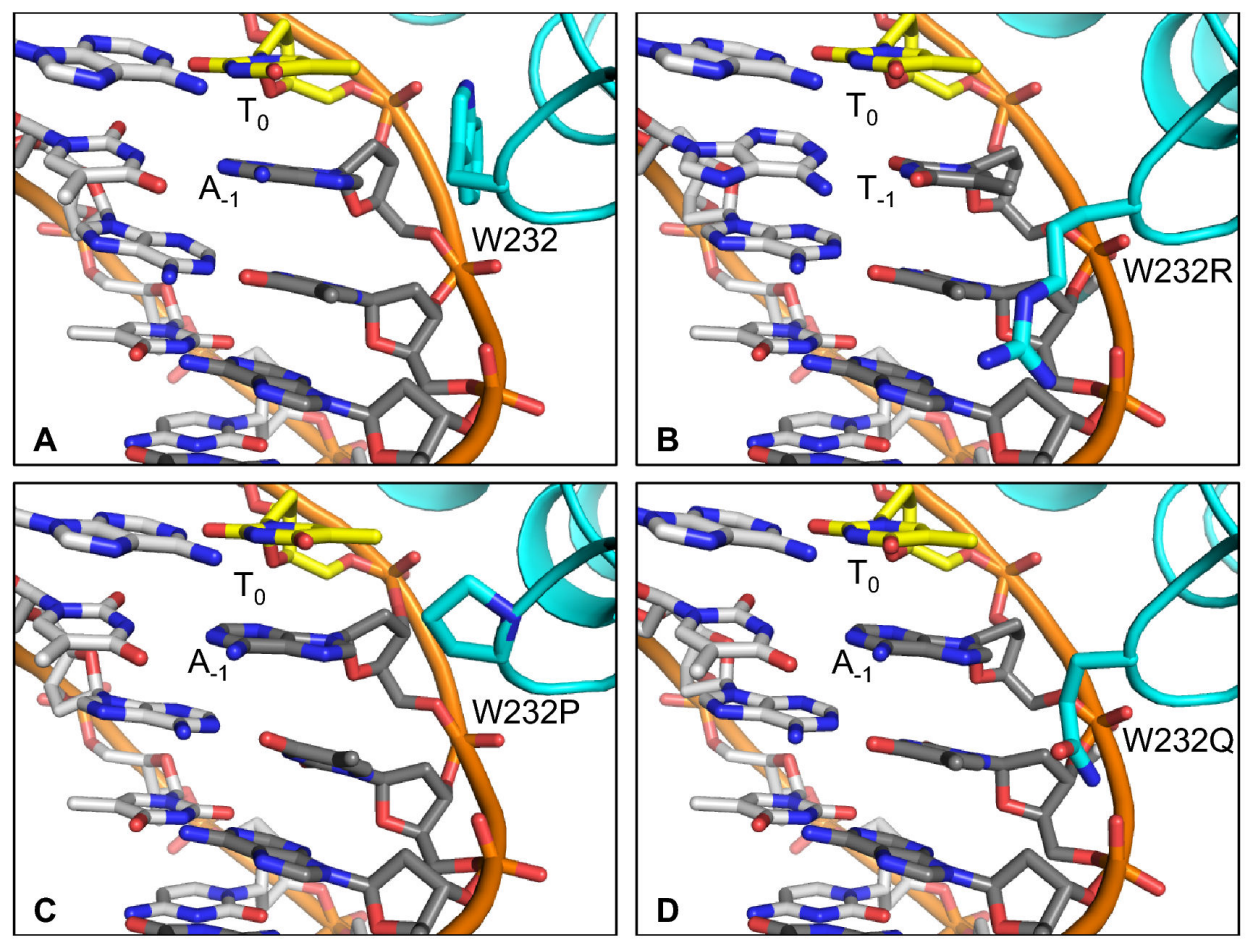
Structural models for representative W232 substitutions. Models were built for all 20 possible amino acids at position 232 with DNA target sites for all 16 nucleotide combinations at positions 0 and -1. Panels A, B, C, and D depict the models for W232, W232R, W232P, and W232Q, respectively. The amino acid at position 232 is labeled using the single letter Code. T_0_ and the nucleotide at position -1 are also labeled. Models shown are of the DNA target with the highest predicted binding affinity for the corresponding W232 substitution, from the flexible backbone simulations. For these substitutions, differences between the fixed and flexible backbone models are slight.

To better understand the context-dependent effects of W232 substitutions on TAL effector activity and specificity, using the solved crystal structure of PthXo1 bound to its target [4], we built structural models for all 20 amino acids at position 232 and calculated binding profiles for each in association with each of 16 DNA targets representing all possible nucleotide combinations at positions 0 and -1 (the base 5’ to position 0 that also makes contact to W232 in the PthXo1 structure). Representative models are depicted in Figure 6. Binding energies for all models are provided in Table S10. We explored two levels of conformational flexibility, either keeping the protein backbone completely fixed or allowing limited flexibility in the neighborhood of the mutation (see Materials and Methods). In the fixed-backbone simulations, W232 in complex with a T at position 0 yielded the highest predicted binding affinity overall (Figure 6A), followed by non-polar substitutions (isoleucine and valine) that maintained packing against the T_0_ methyl group. We did not see a strong dependence of binding energy on position -1, although some substitutions appeared to disfavor T at -1, likely due to steric clashes. We saw a different profile of binding energies in the flexible backbone simulations, with arginine, followed by other polar amino acids (lysine and glutamine), yielding the highest binding affinities, but still generally preferring T at position 0. Examination of the flexible backbone models revealed that contacts to the DNA phosphate backbone predominated, with protein backbone shifts allowing the mutated side chains to form hydrogen bonds to phosphate oxygen atoms (Figure 6B and D). In addition, several of the flexible backbone models, like the solved PthXo1 structure, show the side chain of the amino acid at position 232 in close proximity to, and possibly interacting with base -1 (Figure 6). These contacts were also observed in the fixed backbone models, although they were slightly less pronounced. Overall, both sets of models suggest that portions of the TAL effector N terminus near W232 may influence interaction with the 0^th^ position base and that the neighboring base may also affect 0^th^ position specificity. The models are therefore consistent with our experimental observations that TAL effector activity and specificity depend on CRR (and target) composition and N terminal context.

## Discussion

 We carried out a set of experiments to establish whether the requirement for T in position 0 of TAL effector EBEs indeed depends on W232, as suggested by structural data. In particular, we sought to identify single amino acid substitutions for W232 that would relax or alter position 0 specificity and maintain relatively high levels of activity. Tests of all 19 W232 substitution variants of TAL effector PthXo1 in a GUS reporter assay of TAL effector activity revealed that most substitutions altered specificity yet reduced activity, but some altered specificity while maintaining or increasing activity. When the latter substitutions were tested in a second effector, TAL868, however, the effects were different, revealing that the effects of substitutions on activity and 0^th^ position specificity depend on TAL effector context, specifically the composition of the CRR (or the corresponding RVD-encoded bases of the target). This dependence on CRR composition was also evident in TALENs assayed in a yeast single strand annealing assay, but the TAL effector and TALEN assays themselves conflicted with regard to the changes in specificity and activity that resulted from each substitution, revealing a further effect of structural context or an effect of functional context. Additionally, in EMSAs of DNA binding by TAL868, the substitutions resulted in no detectable change to position 0 base preference, although some reduction of binding affinity to the preferred target (with T at base 0) was observed. So, the changes to activity and specificity observed in the TAL effector and TALEN activity assays were not readily explained in relation to apparent binding affinity. In addition to effects of CRR composition and functional context, we also discovered an influence of N-terminal context on specificity and activity, and on changes due to substitutions at the W232 or equivalent position, by using a chimeric RTL-PthXo1 chimeric construct. The RTL N terminus, which has an arginine (R298) at the position equivalent to W232, changed the base 0 specificity to G, which was different from the specificity for A observed of PthXo1 with the W232R substitution. Further, substitutions at R298, even R298W, abolished activity. Our results provide the first experimental evidence that W232 encodes the requirement for T_0_ in TAL effector binding sites. They also reveal unexpected complexity, with the activity and 0^th^ position specificity of W232 substitution variants depending on TAL effector CRR and target composition, on whether TAL effector activity, TALEN activity, or DNA binding is assayed, and on the N-terminal sequence context of the substitutions.

Dependence of the effects of W232 substitutions on the CRR may relate to differences throughout the CRR (or target), or to differences near the cryptic -1^st^ and 0^th^ repeats. Recent evidence has shown that different RVDs have different relative contributions to apparent affinity [41,42]. It is possible that different content and distributions of strong- and weak-binding RVDs throughout the CRR could differentially affect the importance of strong interactions between the N terminus (including W232) and position 0 of the EBE and thus allow more flexibility in the N terminal region in some instances that could result in different effects of the same substitution at W232. One model of TAL effector DNA binding proposes that the N terminal region with the cryptic repeats nucleates interaction with the DNA and that consecutive RVD-nucleotide matches are responsible for target acquisition [43] In this scenario, differences particularly at the N terminal end of the CRR could differentially affect the relative importance of interactions of the N-terminus with the DNA. Or, the effect could be directly on the cryptic repeat conformations through interactions at the interface with the different proximal CRR repeats, which might have slightly different conformations depending on their RVDs. Differences in RVD and target site composition, whether throughout the CRR or primarily at its N-terminal end, could also cause differential effects on TAL effector backbone conformation that through intramolecular forces subtly reshape the interaction of the N terminus with position 0. As noted, in our TAL effector activity assay we used the native PthXo1 CRR and its natural EBE, and the alignment of these contains several mismatches. In contrast, TAL868 is an artificial TAL effector and it was assayed on its perfect-match EBE. Therefore the differential effects discussed here may have been amplified by the differences in matching. A recent study found that the activity of TAL effector AvrBs3 on a naturally occurring EBE that has several mismatches was more sensitive to nucleotide substitutions for the 0^th^ position T than an artificially assembled TAL effector engineered to exactly match that EBE [42]. Furthermore, the fact that TAL868 (16 RVDs) is shorter than PthXo1 (24 RVDs) might have contributed to the differential effects. One might expect a shorter TAL effector to be less tolerant of mismatches generally, including at base 0. The results for the unsubstituted TAL868 vs. PthXo1 in the TAL effector assay are consistent with this. However, the correlation breaks down when all of the W232 substitution variants are considered (Figure 2). And in the TALEN assay (Figure 3), the unsubstituted TAL868 appears to be more tolerant to base 0 mismatches than PthXo1.

Regarding differences observed among the different types of assays we carried out, TAL effector activity, TALEN activity, and protein-DNA binding, several factors might provide explanation. First, despite careful standardization of conditions and leaf age, we found the TAL effector activity assay to be highly variable, both within experiments (across replicates) and across experiments performed on different days. Although we accounted for this inherent variability by using a mixed linear model to separate treatment effects on activity from effects of other variables, and determine significance, the variability may have introduced artifacts. Another factor that may explain the differing results is the differing construct architectures used in the different assays. While all proteins contained all four cryptic repeats that constitute the well ordered portion of the N terminus involved in DNA binding [40], the TAL effector assay used a full-length TAL effector, and the TALEN assays and EMSAs used a TAL effector partially truncated at the N and C termini. Additionally, the TALEN constructs contained a C-terminal *Fok*I catalytic domain fusion and an added N-terminal nuclear localization signal sequence. W232 substitutions may differentially affect the rates at which these slightly different proteins bind to and release their DNA targets, affecting activity (or affinity) differently. Or, the substitutions may cause different shifts in structure in the different architectures, differentially affecting specificity for base 0. Conversely, similar changes in structure could have differential effects on the different assay readouts. For example, a specific substitution might alter the interaction of the TAL effector with the host transcriptional machinery, but have no effect on DNA binding, or in the TALEN context, on DNA cleavage. A third factor that might explain the conflicting results across assays might be differences in EBE context, especially the base at position -1 (directly 5’ to position 0). This base contacts W232 in the bound PthXo1 structure, and could conceivably affect the behavior of substitutions there [4]. Based on the lack of conservation at base -1 of natural EBEs and extensive reported success in targeting without regard for base -1 identity, we neither anticipated nor controlled for the possibility of an effect of base -1 on the behavior of W232 substitutions, and there were indeed differences across our assays. In the TAL effector activity assays, base -1 was a G. In the TALEN assay, one of the paired EBEs was preceded by C at position -1, while the other was preceded by a G. In the EMSA, the -1 base was a C. 

The effects of N-terminal context we observed by examining the RTL-PthXo1 chimera support a number of the models we have discussed above to explain differential effects of W232 substitutions in different contexts. Because the preference of the chimera for G at the 0^th^ position differed from the preference for A of PthXo1 with the W232R substitution (matching R298 of the RTL), and the overall effects of substitutions in these two proteins differed, even though residues to either side of W232 and R298 are conserved, global structural deviations in the cryptic repeats that presumably exist due to sequence differences scattered throughout the N terminal region appear to influence the role of the W232/R298 position in base 0 specificity. Further support for this conclusion is provided by the crystal structure of bound PthXo1 in which the 0^th^ and -1^st^ repeats pack tightly together, with the interhelical loop of the 0^th^ repeat also entering the major groove near T_0_ [4]. This juxtaposition suggests that the 0^th^ repeat, possibly influenced in turn by other portions of the N-terminus, may be involved in positioning repeat -1 or stabilizing its interaction with base 0. The inference that differences in overall N terminal conformation between the RTL and PthXo1 result in the different specificities for base 0, and in the different results of substitutions observed, is consistent with shifts in cryptic repeat conformation caused by differences in CRRs or overall architecture (discussed above) resulting in differential effects as well. Also, the fact that RTL-PthXo1 had nearly 10 fold lower activity than PthXo1 could reflect incompatibility between the respective portions of these two proteins, again hinting at intramolecular dependencies. These could be direct, such as physical interactions at the interface of the CRR and the cryptic repeats, or indirect, such as effects on conformation of one portion that are linked though the backbone to forces exerted by the other.

In all, our results indicate that engineering TAL effectors to have robust, altered specificities will require changes in addition to or instead of substitutions for W232, likely at other positions in the cryptic -1st and in the 0th repeat, in other portions of TAL effector N terminus, and possibly at the interface of the cryptic repeats with the repeats of the CRR. The consistency of the native W232 preference for T at base 0, across TAL effectors and irrespective of whether TAL effector activity, TALEN activity, or TAL effector DNA binding is measured, compared to the inconsistent effects of substitutions at W232, suggests that the native tryptophan results in the most stable N terminal conformation. Indeed, the sensitivity of the specificity and activity of the substitution variants to context might be explained by their rendering the cryptic repeat region structurally less stable and subject to subtle, context-dependent shifts. The fact that substitutions for R298 in the RTL N-terminus completely abolished activity of the chimeric RTL-PthXo1 protein is consistent with this idea, suggesting that in that context, the substitutions might be even more destabilizing to the N-terminus (importantly, each of the substituted TAL868 substitution variants used in the EMSA, like the unsubstituted protein, expressed well and stably, indicating that the substitutions likely do not affect overall protein integrity). The results of our structural modeling are also consistent, showing that tryptophan at position 232, or arginine in the flexible backbone simulation, in combination with T_0_ yields the most favorable predicted binding energy. The modeling results may in fact indicate the reason these residues are strictly conserved across TAL effectors and RTLs, respectively: i.e., that they confer the most stable structures and best possible affinities for the targets. More solved TAL effector crystal structures, including structures of some of the substitution variants studied here, and RTL structures, will shed light on the apparently complex picture of the role of W232 and the cryptic repeats in the preference for T_0_ and in overall DNA binding. Such insight will help to guide future efforts to engineer TAL effectors with broadened DNA targeting capacity.

While this manuscript was under review, Lamb and colleagues [44] published a related study that corroborates our findings. They reported that directed evolution of TAL effector N-terminal domains with altered specificities for the 0^th^ position nucleotide and robust activity involved mutations of W232 and the surrounding amino acids (K230-G234), supporting our conclusion of an apparently complex requirement for the contextual residues of W232. In accordance with our work and the finding for RTLs [30], they found that arginine at position 232 confers specificity for G at the 0^th^ position, but again, robust activity depended on additional substitutions for surrounding residues. Lamb and colleagues also recovered variants with robust activity on targets with any base at the 0^th^ position (relaxed specificity) but none specific for targets preceded by A or by C. This agrees with our notion that designing such specificities may require wholesale re-engineering of both the -1^st^ and 0^th^ cryptic repeats. The authors did not directly assess the effect of different RVD sequences. They did use different assays, but they did not observe as dramatic an effect of assay used as we did. They obtained relatively consistent (but not perfectly correlated) results across a TAL effector-VP64 transactivation assay, a TAL effector recombinase activity assay, and a TALEN assay, each in cultured human embryonic kidney cells, and an *in vitro* DNA binding assay using a TAL effector-maltose binding protein fusion. The reason for this difference from our results may be inherent to the assays and cell types, or to differences in the TAL effector RVD sequences or scaffolds used.

## Supporting Information

Data File S1 **All raw data used in mixed linear model analysis.**
(CSV)

Figure S1 **Purified TAL868 (W232) and amino acid substitution variants separated by SDS-PAGE and visualized by Coomassie blue staining.**
(PDF)

Figure S2 **Activity of PthXo1 variants with all 19 possible single amino acid substitutions for W232.** Activity was measured in an *Agrobacterium*-mediated transient expression assay in *Nicotiana benthamiana* leaves, using a GUS reporter gene cloned downstream of a minimal promoter (see Materials and Methods) containing the PthXo1 effector binding element (EBE) with the 0^th^ position thymine (EBE_PthXo1-T), or variants with adenine, cytosine, or guanine as base 0 (EBE_PthXo1-A, EBE_PthXo1-C, and EBE_PthXo1-G, and respectively). Horizontal lines at the bottom group treatments that were tested on the same day in the same experiment. To facilitate comparison across multiple experiments, activity was normalized to the activity of PthXo1 with EBE_PthXo1-T, which was included in each experiment (but shown only for the first experiment) and set to 1.0. None, no TAL effector. Values are the mean of three replicates. Error bars represent s.d.
(PDF)

Figure S3 **Alignment of N terminal sequences of *Ralstonia* TAL-like effectors (RTLs) and PthXo1.** RTL sequences are those in GenBank with complete N-terminal and central repeat region sequences, RSc1815 (GenBank ID CAD15517.1) from *R. solanacearum* strain GMI1000, Hpx17 (GenBank ID AB178011.1) from strain RS1085, and RscCAQ18687 (GenBank ID CAQ18687.1) from strain MolK2. PthXo1 (GenBank ID ACD58243.1) is from *Xanthomonas oryzae* strain PXO99^A^. Residues corresponding approximately to the -1^st^ repeat are highlighted in light grey. Residues corresponding approximately to the 0^th^ repeat are highlighted in darker grey. W232 and aligned arginine residues are shown in large bold type. Sequences were aligned using ClustalW [45].
(PDF)

Figure S4 **Secondary structure predictions for PthXo1 and Rsc1815 N-terminal regions.** Left, PthXo1 (GenBank accession ACD58243.1) from *Xanthomonas oryzae* strain PXO99^A^. Right, RSc1815 (GenBank accession CAD15517.1) from *Ralstonia solanacearum* strain GMI1000. Secondary structures were predicted using Psipred [46].
(PDF)

Script S1 **Example R code for Figure 2 and for Tables S5-S7.**
(TXT)

Table S1 **Oligonucleotides used for pAH236 W232 substitutions.**
(PDF)

Table S2 **Oligonucleotides used for RTL-PthXo1 R298 substitutions.**
(PDF)

Table S3 **Oligonucleotides used for GUS reporter constructs.**
(PDF)

Table S4 **Oligonucleotides used for W232 substitutions in TALEN yeast expression vectors.**
(PDF)

Table S5 **Differences in size of effects on GUS activity between W232 substitutions co-delivered with targets with 0^th^ A, C, G, or T in position 0 and the corresponding negative control.**
(PDF)

Table S6 **Differences in size of effects on GUS activity between W232 substitution variants co-delivered with targets with 0^th^ position A, C, G, or T, and the wild-type TAL effector co-delivered with the target with 0^th^ position T.**
(PDF)

Table S7 **Differences in size of effects on GUS activity between W232 substitution variants co-delivered with targets with 0th position A, C, or G, and the same TAL effector co-delivered with the target with 0^th^ position T, used to determine specificity for the 0^th^ position.**
(PDF)

Table S8 **Statistical significance of values shown in Figure 3, “Activity of TALENs with selected single amino acid substitutions for W232 on targets with A, C, G, or T at the 0^th^ position.”.**
(PDF)

Table S9 **Statistical significance of values shown in Figure 5, “Activity of chimeric TAL effector RTL-PthXo1 with single amino acid substitutions for R298 on targets with A, C, G, or T at the 0th position.”.**
(PDF)

Table S10 **Binding energies for structural models generated using the Rosetta molecular modeling software package.**
(CSV)

## References

[1] Doyle EL, Stoddard BL, Voytas DF, Bogdanove AJ (2013) TAL effectors: highly adaptable phytobacterial virulence factors and readily engineered DNA-targeting proteins. Trends Cell Biol (. (2013)) doi:10.1016/j.tcb.2013.1004.1003. PubMed: 23707478. PMC3729746 23707478

[2] Moscou MJ, Bogdanove AJ (2009) A simple cipher governs DNA recognition by TAL effectors. Science 326: 1501. doi:10.1126/science.1178817. PubMed: 19933106. 19933106

[3] Boch J, Scholze H, Schornack S, Landgraf A, Hahn S et al. (2009) Breaking the code of DNA binding specificity of TAL-type III effectors. Science 326: 1509-1512. doi:10.1126/science.1178811. PubMed: 19933107. 19933107

[4] Mak AN-S, Bradley P, Cernadas RA, Bogdanove AJ, Stoddard BL (2012) The crystal structure of TAL effector PthXo1 bound to its DNA target. Science 335: 716-719. doi:10.1126/science.1216211. PubMed: 22223736. 22223736 PMC3427646

[5] Deng D, Yan C, Pan X, Mahfouz M, Wang J et al. (2012) Structural basis for sequence-specific recognition of DNA by TAL effectors. Science 335: 720-723. doi:10.1126/science.1215670. PubMed: 22223738. 22223738 PMC3586824

[6] Cermak T, Doyle EL, Christian M, Wang L, Zhang Y et al. (2011) Efficient design and assembly of custom TALEN and other TAL effector-based constructs for DNA targeting. Nucleic Acids Res 39: 7879. doi:10.1093/nar/gkr739. PubMed: 21493687. PMC3130291 21493687

[7] Miller JC, Tan S, Qiao G, Barlow KA, Wang J et al. (2011) A TALE nuclease architecture for efficient genome editing. Nat Biotechnol 29: 143-148. PubMed: 21179091. 21179091 10.1038/nbt.1755

[8] Zhang F, Cong L, Lodato S, Kosuri S, Church GM et al. (2011) Efficient construction of sequence-specific TAL effectors for modulating mammalian transcription. Nat Biotechnol 29: 149-153. doi:10.1038/nbt.1775. PubMed: 21248753. 21248753 PMC3084533

[9] Morbitzer R, Römer P, Boch J, Lahaye T (2010) Regulation of selected genome loci using *de* *novo*-engineered transcription activator-like effector (TALE)-type transcription factors. Proceedings of the National Academy of Sciences of the USA 107: 21617-21622. doi:10.1073/pnas.1013133107. PubMed: 21106758. 21106758 PMC3003021

[10] Geissler R, Scholze H, Hahn S, Streubel J, Bonas U et al. (2011) Transcriptional activators of human genes with programmable DNA-specificity. PLOS ONE 6: e19509. doi:10.1371/journal.pone.0019509. PubMed: 21625585. 21625585 PMC3098229

[11] Mahfouz MM, Li L, Piatek M, Fang X, Mansour H et al. (2012) Targeted transcriptional repression using a chimeric TALE-SRDX repressor protein. Plant Mol Biol 78: 311-321. doi:10.1007/s11103-011-9866-x. PubMed: 22167390. 22167390 PMC3259320

[12] Blount BA, Weenink T, Vasylechko S, Ellis T (2012) Rational diversification of a promoter providing fine-tuned expression and orthogonal regulation for synthetic biology. PLOS ONE 7: e33279. doi:10.1371/journal.pone.0033279. PubMed: 22442681. 22442681 PMC3307721

[13] Cong L, Zhou R, Kuo Y-c, Cunniff M, Zhang F (2012) Comprehensive interrogation of natural TALE DNA-binding modules and transcriptional repressor domains. Nature Commun 3: 968. doi:10.1038/ncomms1962. PubMed: 22828628. 22828628 PMC3556390

[14] Christian M, Cermak T, Doyle EL, Schmidt C, Zhang F et al. (2010) Targeting DNA double-strand breaks with TAL effector nucleases. Genetics 186: 757-761. doi:10.1534/genetics.110.120717. PubMed: 20660643. 20660643 PMC2942870

[15] Mahfouz MM, Li L, Shamimuzzaman M, Wibowo A, Fang X et al. (2011) *De* *novo*-engineered transcription activator-like effector (TALE) hybrid nuclease with novel DNA binding specificity creates double-strand breaks. Proc Natl Acad Sci U S A 108: 2623-2628. doi:10.1073/pnas.1019533108. PubMed: 21262818. 21262818 PMC3038751

[16] Li T, Huang S, Jiang WZ, Wright D, Spalding MH et al. (2011) TAL nucleases (TALNs): hybrid proteins composed of TAL effectors and *Fok*I DNA-cleavage domain. Nucleic Acids Res 39: 359-372. doi:10.1093/nar/gkq704. PubMed: 20699274. 20699274 PMC3017587

[17] Hartlerode AJ, Scully R (2009) Mechanisms of double-strand break repair in somatic mammalian cells. Biochem J 423: 157-168. doi:10.1042/BJ20090942. PubMed: 19772495. 19772495 PMC2983087

[18] Puchta H (2005) The repair of double-strand breaks in plants: mechanisms and consequences for genome evolution. J Exp Bot 56: 1-14. doi:10.1093/jxb/eri123. PubMed: 15557293. 15557293

[19] Kanaar R, Hoeijmakers JH, van Gent DC (1998) Molecular mechanisms of DNA double strand break repair. Trends Cell Biol 8: 483-489. doi:10.1016/S0962-8924(98)01383-X. PubMed: 9861670. 9861670

[20] Mussolino C, Cathomen T (2012) TALE nucleases: tailored genome engineering made easy. Curr Opin Biotechnol 23: 644-650. doi:10.1016/j.copbio.2012.01.013. PubMed: 22342754. 22342754

[21] Römer P, Recht S, Strauss T, Elsaesser J, Schornack S et al. (2010) Promoter elements of rice susceptibility genes are bound and activated by specific TAL effectors from the bacterial blight pathogen, *Xanthomonas* *oryzae* pv. oryzae. New Phytol 187: 1048-1057. doi:10.1111/j.1469-8137.2010.03217.x. PubMed: 20345643. 20345643

[22] Römer P, Strauss T, Hahn S, Scholze H, Morbitzer R et al. (2009) Recognition of AvrBs3-like proteins is mediated by specific binding to promoters of matching pepper *Bs3* alleles. Plant Physiol 150: 1697-1712. doi:10.1104/pp.109.139931. PubMed: 19448036. 19448036 PMC2719119

[23] Yu Y, Streubel J, Balzergue S, Champion A, Boch J, et al. (2011) Colonization of rice leaf blades by an African strain of *Xanthomonas* *oryzae* pv. oryzae depends on a new TAL effector which induces the rice nodulin-3 *Os11N3* gene. Mol Plant-Microbe Interact 24: 1102-1113 10.1094/MPMI-11-10-0254 21679014

[24] Briggs AW, Rios X, Chari R, Yang L, Zhang F et al. (2012) Iterative capped assembly: rapid and scalable synthesis of repeat-module DNA such as TAL effectors from individual monomers. Nucleic Acids Res 40: e117. doi:10.1093/nar/gks608. PubMed: 22740649. 22740649 PMC3424587

[25] Sun N, Liang J, Abil Z, Zhao H (2012) Optimized TAL effector nucleases (TALENs) for use in treatment of sickle cell disease. Mol Biosyst 8: 1255-1263. doi:10.1039/c2mb05461b. PubMed: 22301904. 22301904

[26] Gao H, Wu X, Chai J, Han Z (2012) Crystal structure of a TALE protein reveals an extended N-terminal DNA binding region. Cell Res 22: 1716-1720. doi:10.1038/cr.2012.156. PubMed: 23147789. 23147789 PMC3515758

[27] Mussolino C, Morbitzer R, Lütge F, Dannemann N, Lahaye T et al. (2011) A novel TALE nuclease scaffold enables high genome editing activity in combination with low toxicity. Nucleic Acids Res 39: 9283-9293. doi:10.1093/nar/gkr597. PubMed: 21813459. 21813459 PMC3241638

[28] Salanoubat M, Genin S, Artiguenave F, Gouzy J, Mangenot S et al. (2002) Genome sequence of the plant pathogen *Ralstonia* *solanacearum* . Nature 415: 497-502. doi:10.1038/415497a. PubMed: 11823852. 11823852

[29] Genin S, Denny TP (2012) Pathogenomics of the *Ralstonia* *solanacearum* species complex. Annu Rev Phytopathol 50: 67-89. doi:10.1146/annurev-phyto-081211-173000. PubMed: 22559068. 22559068

[30] De Lange O, Schreiber T, Schandry N, Radeck J, Braun KH et al. (2013) Breaking the DNA binding code of *Ralstonia* *solanacearum* TAL effectors provides new possibilities to generate plant resistance genes against bacterial wilt disease. New Phytol 199: 773–786. doi:10.1111/nph.12324. PubMed: 23692030. 23692030

[31] Verdier V, Triplett LR, Hummel AW, Corral R, Cernadas RA et al. (2012) Transcription activator-like (TAL) effectors targeting *OsSWEET* genes enhance virulence on diverse rice (*Oryza* *sativa*) varieties when expressed individually in a TAL effector-deficient strain of *Xanthomonas* *oryzae* . New Phytol 196: 1197-1207. doi:10.1111/j.1469-8137.2012.04367.x. PubMed: 23078195. 23078195

[32] Cermak T, Doyle EL, Christian M, Wang L, Zhang Y et al. (2011) Efficient design and assembly of custom TALEN and other TAL effector-based constructs for DNA targeting. Nucleic Acids Res 39: e82. doi:10.1093/nar/gkr218. PubMed: 21493687. 21493687 PMC3130291

[33] Nakagawa T, Kurose T, Hino T, Tanaka K, Kawamukai M et al. (2007) Development of series of gateway binary vectors, pGWBs, for realizing efficient construction of fusion genes for plant transformation. J Biosci Bioeng 104: 34-41. doi:10.1263/jbb.104.34. PubMed: 17697981. 17697981

[34] Kay S, Hahn S, Marois E, Hause G, Bonas U (2007) A bacterial effector acts as a plant transcription factor and induces a cell size regulator. Science 318: 648-651. doi:10.1126/science.1144956. PubMed: 17962565. 17962565

[35] Townsend JA, Wright DA, Winfrey RJ, Fu F, Maeder ML et al. (2009) High-frequency modification of plant genes using engineered zinc-finger nucleases. Nature 459: 442-445. doi:10.1038/nature07845. PubMed: 19404258. 19404258 PMC2743854

[36] Zhang Y, Zhang F, Li X, Baller JA, Qi Y et al. (2013) Transcription activator-like effector nucleases enable efficient plant genome engineering. Plant Physiol 161: 20-27. doi:10.1104/pp.112.205179. PubMed: 23124327. 23124327 PMC3532252

[37] Leaver-Fay A, Tyka M, Lewis SM, Lange OF, Thompson J, et al. (2011) Chapter nineteen - Rosetta3: An object-oriented software suite for the simulation and design of macromolecules. In: Michael LJ Ludwig B Methods Enzymol: Academic Press . pp. 545-574 10.1016/B978-0-12-381270-4.00019-6 PMC4083816 21187238

[38] Römer P, Recht S, Strauss T, Elsaesser J, Schornack S et al. (2010) Promoter elements of rice susceptibility genes are bound and activated by specific TAL effectors from the bacterial blight pathogen, Xanthomonas oryzae pv. oryzae. New Phytol 187: 1048-1057. doi:10.1111/j.1469-8137.2010.03217.x. PubMed: 20345643. 20345643

[39] Yang B, Sugio A, White FF (2006) *Os8N3* is a host disease-susceptibility gene for bacterial blight of rice. Proc Natl Acad Sci U S A 103: 10503-10508. doi:10.1073/pnas.0604088103. PubMed: 16798873. 16798873 PMC1502487

[40] Gao H, Wu X, Chai J, Han Z (2012) Crystal structure of a TALE protein reveals an extended N-terminal DNA binding region. Cell Res 22: 1716-1720. doi:10.1038/cr.2012.156. PubMed: 23147789. 23147789 PMC3515758

[41] Streubel J, Blücher C, Landgraf A, Boch J (2012) TAL effector RVD specificities and efficiencies. Nat Biotechnol 30: 593-595. doi:10.1038/nbt.2304. PubMed: 22781676. 22781676

[42] Meckler JF, Bhakta MS, Kim MS, Ovadia R, Habrian CH et al. (2013) Quantitative analysis of TALE-DNA interactions suggests polarity effects. Nucleic Acids Res 41: 4118-4128. doi:10.1093/nar/gkt085. PubMed: 23408851. 23408851 PMC3627578

[43] Mak AN-S, Bradley P, Bogdanove AJ, Stoddard BL (2013) TAL effectors: function, structure, engineering and applications. Curr Opin Struct Biol 23: 93-99. PubMed: 23265998. 23265998 10.1016/j.sbi.2012.11.001 PMC3572262

[44] Lamb BM, Mercer AC, Barbas CF 3rd (2013) Directed evolution of the TALE N-terminal domain for recognition of all 5' bases. Nucleic Acids Res (. (2013)) doi:10.1093/nar/gkt1754. PubMed: 23980031. PMC3834825 23980031

[45] Larkin MA, Blackshields G, Brown NP, Chenna R, McGettigan PA et al. (2007) Clustal W and Clustal X version 2.0. Bioinformatics 23: 2947-2948. doi:10.1093/bioinformatics/btm404. PubMed: 17846036. 17846036

[46] Buchan DWA, Ward SM, Lobley AE, Nugent TCO, Bryson K et al. (2010) Protein annotation and modelling servers at University College London. Nucleic Acids Res 38: W563-W568. doi:10.1093/nar/gkq427. PubMed: 20507913. 20507913 PMC2896093

