# Supplementary material for: TAL Effector Specificity for base 0 of the DNA Target Is Altered in a Complex, Effector- and Assay-Dependent Manner by Substitutions for the Tryptophan in Cryptic Repeat –1
Source: PLoS One. 2013 Dec 3;8(12):e82120. doi: 10.1371/journal.pone.0082120 (PMC3849474; doi:10.1371/journal.pone.0082120)
Supplement: Figure S1 — Purified TAL868 (W232) and amino acid substitution variants separated by SDS-PAGE and visualized by Coomassie blue staining. (PDF) [file pone.0082120.s002.pdf]

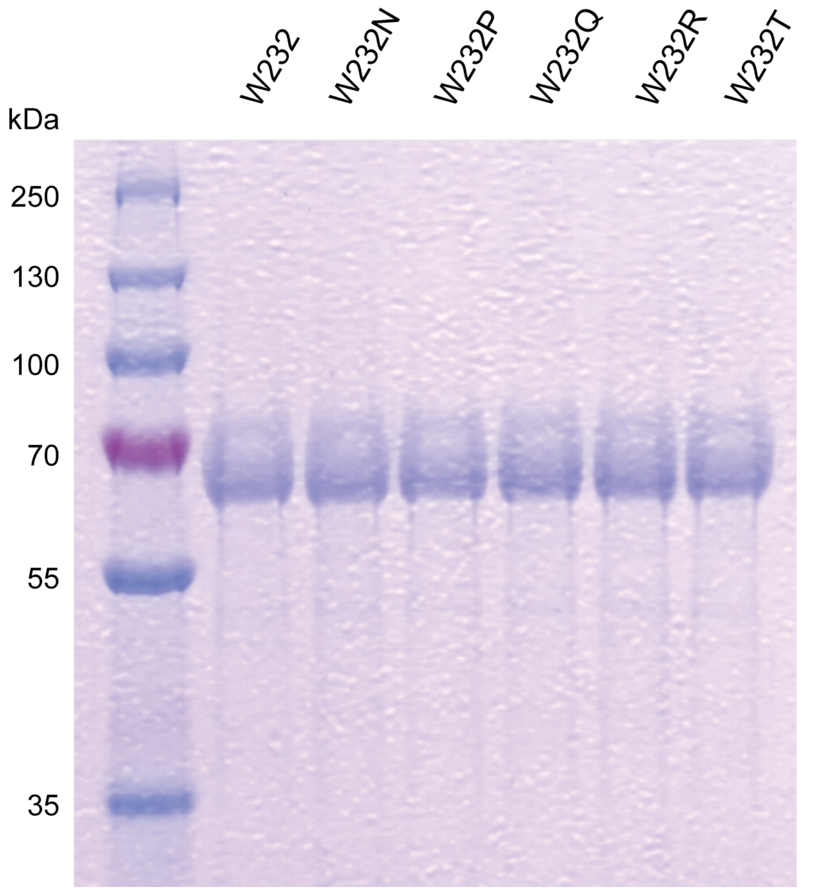

Figure S1. Purified TAL868 (W232) and amino acid substitution variants separated by SDS PAGE and visualized by Coomassie blue staining.
