# Supplementary material for: TAL Effector Specificity for base 0 of the DNA Target Is Altered in a Complex, Effector- and Assay-Dependent Manner by Substitutions for the Tryptophan in Cryptic Repeat –1
Source: PLoS One. 2013 Dec 3;8(12):e82120. doi: 10.1371/journal.pone.0082120 (PMC3849474; doi:10.1371/journal.pone.0082120)
Supplement: Figure S2 — Activity of PthXo1 variants with all 19 possible single amino acid substitutions for W232. Activity was measured in an Agrobacterium-mediated transient expression assay in Nicotiana benthamiana leaves, using a GUS reporter gene cloned downstream of a minimal promoter (see Materials and Methods) containing the PthXo1 effector binding element (EBE) with the 0th position thymine (EBE_PthXo1-T), or variants with adenine, cytosine, or guanine as base 0 (EBE_PthXo1-A, EBE_PthXo1-C, and EBE_PthXo1-G, and respectively). Horizontal lines at the bottom group treatments that were tested on the same day in the same experiment. To facilitate comparison across multiple experiments, activity was normalized to the activity of PthXo1 with EBE_PthXo1-T, which was included in each experiment (but shown only for the first experiment) and set to 1.0. None, no TAL effector. Values are the mean of three replicates. Error bars represent s.d. (PDF) [file pone.0082120.s003.pdf]

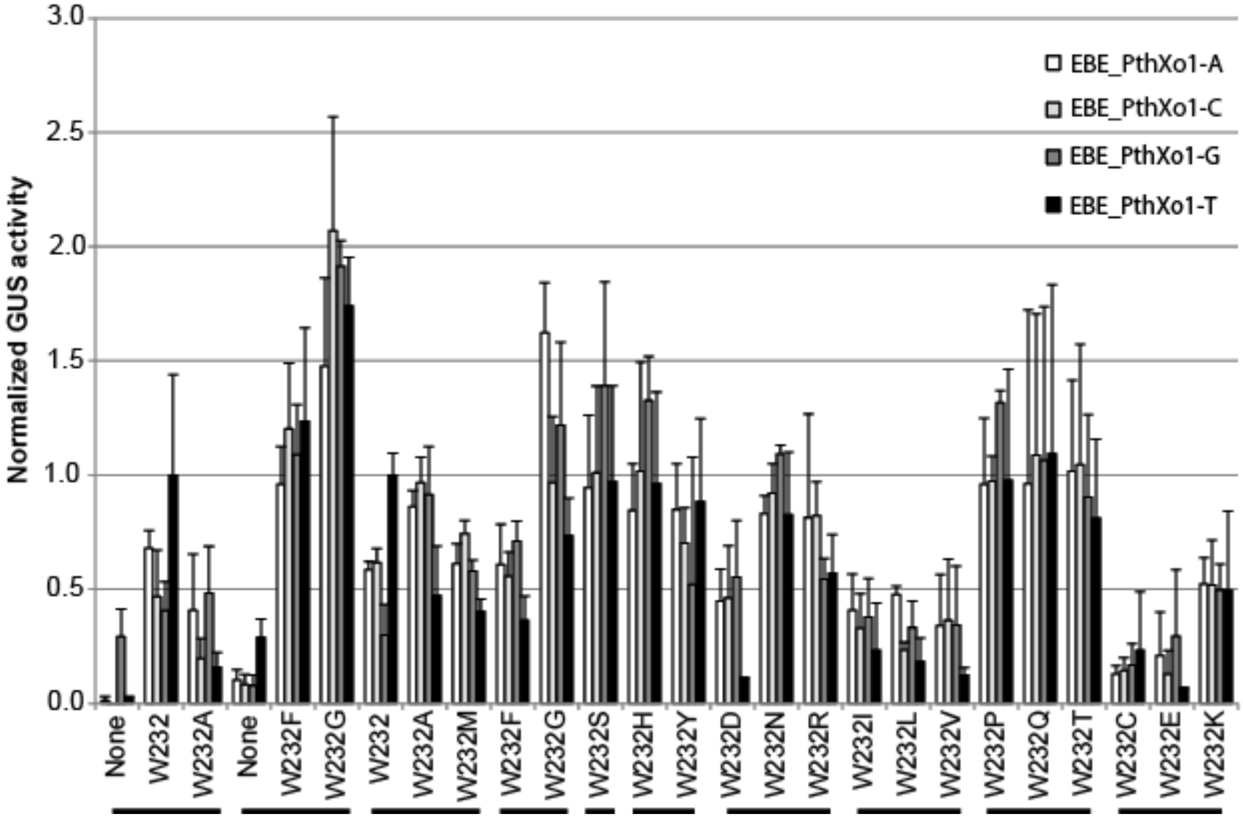

Figure S2. Activity of PthXo1 variants with all 19 possible single amino acid substitutions for W232. Activity was measured in an *Agrobacterium*-mediated transient expression assay in *Nicotiana benthamiana* leaves, using a GUS reporter gene cloned downstream of a minimal promoter (see Materials and Methods) containing the PthXo1 effector binding element (EBE) with the 0th position thymine (EBE_PthXo1-T), or variants with adenine, cytosine, or guanine as base 0 (EBE_PthXo1-A, EBE_PthXo1-C, and EBE_PthXo1-G, and respectively). Horizontal lines at the bottom group treatments that were tested on the same day in the same experiment. To facilitate comparison across multiple experiments, activity was normalized to the activity of PthXo1 with EBE_PthXo1-T, which was included in each experiment (but shown only for the first experiment) and set to 1.0. None, no TAL effector. Values are the means of three replicates. Error bars represent s.d.
