# Supplementary material for: TAL Effector Specificity for base 0 of the DNA Target Is Altered in a Complex, Effector- and Assay-Dependent Manner by Substitutions for the Tryptophan in Cryptic Repeat –1
Source: PLoS One. 2013 Dec 3;8(12):e82120. doi: 10.1371/journal.pone.0082120 (PMC3849474; doi:10.1371/journal.pone.0082120)
Supplement: Figure S3 — Alignment of N terminal sequences of Ralstonia TAL-like effectors (RTLs) and PthXo1. RTL sequences are those in GenBank with complete N-terminal and central repeat region sequences, RSc1815 (GenBank ID CAD15517.1) from R. solanacearum strain GMI1000, Hpx17 (GenBank ID AB178011.1) from strain RS1085, and RscCAQ18687 (GenBank ID CAQ18687.1) from strain MolK2. PthXo1 (GenBank ID ACD58243.1) is from Xanthomonas oryzae strain PXO99A. Residues corresponding approximately to the -1st repeat are highlighted in light grey. Residues corresponding approximately to the 0th repeat are highlighted in darker grey. W232 and aligned arginine residues are shown in large bold type. Sequences were aligned using ClustalW [45]. (PDF) [file pone.0082120.s004.pdf]

```
CLUSTAL 2.1 multiple sequence alignment


RSc1815         MRIGKSSGWLNESVSLEYEHVSPPTRPRDTRRRPRAAGDGGLAHLHRRLAVGYAEDTPRT 60
hpx17           MRIGKSSGWLNESVSLEYEHVSPPTRPRDTRRRPRAASDGGLAHLHRRLAVGYAEDTPRT 60
RScCAQ18687     ---------------------------------------------MRRRTAVGRVPGASRS 16
PthXo1          ------------------------------------------------------------


RSc1815         EARSPAPRRPLPVAPASAPPAPSLVPEPPMPVSLPAVSSPRFSAGSSAAITDPFPSLP-- 118
hpx17           GARSPAPRRPLPVAPASAPPAPSLVPEPPMPVSLPVVSSPRFSAGSSAAITDPFSSLP-- 118
RScCAQ18687     GTSPLVLSQPLSRVSASPQPARS-----------SAISSPNFSAGNPTAFANPSPSLPPT 65
PthXo1          ------------------------------------------------------------


RSc1815         ----PTPVLYAMARELEALSDATWQPAVPLPAEPPTDARRGNTVFDEASASSPVIASACP 174
hpx17           ----PTPVLYAMARELKALSDATWQPAVPLPAEPPTDARRGNTVFDEASASSPVIASACP 174
RScCAQ18687     PVLPPTPVLYAMARELEELHNATWQPAVPLTAEPLADARRDNTVVDGGSGSSPAIASARP 125
PthXo1          -----------------------MQSGLRAADDPPPTVR-------------VAVTAAR- 23
                                        *..:  . :* . .*              .:::*

RSc1815         QAFASPPRAPRSARARRARTGGDAWPAPTFLS-RPSSS-------RIGRDVFGKLVALGY 226
hpx17           QAFASPPRAPRSARARRARTGGDAWPAPTFLS-RPSSS-------RIGRDVFGKLVALGY 226
RScCAQ18687     QAFAGPPRAPRSARARRARTAGDAWPAPAYLGPSPSPSPSPSPSPRIAPDLSGKLAALGY 185
PthXo1          -----PPRAKPAPRRRAAQPS-DASPA-----------------------AQVDLRTLGY 54
                     ****  :.* * *:.. ** **                          .* :***

RSc1815         SREQIRKLKQESLSEIAKYHTTLTGQGFTHADICRISRRRQSLRVVARNYPELAAALPEL 286
hpx17           SREQIRKLKQESLSEIAKYHTTLTGQGFTHADICRISRRRQSLRVVARNYPELAAALPEL 286
RScCAQ18687     SREQIRKLKQESLAEVAKYHATLAGQGFTHADICRISRRWQSLRVVANNYPELMAALPRL 245
PthXo1          SQQQQEKIKPKVGSTVAQHHEALVGHGFTHAHIVALSRHPAALGTVAVKYQDMIAALPEA 114
                *::* .*:* :  : :*::* :*.*:*****.*  :**:  :* .** :* :: ****.

RSc1815         TRAHIVDIARQRSGDLALQALLPVATALTAAPLRLSASQIATVAQYGERPAIQALYRLRR 346
hpx17           TRAHIVDIARQRSGDLALQALLPVATALTAAPLRLSASQIATVAQYGERPAIQALYRLRR 346
RScCAQ18687     TTAQIVDIARQRSGDLALQALLPVAAALTAAPLGLSASQIATVAQYGERPAIQALYRLRR 305
PthXo1          THEDIVGVGKQWSGARALEALLTVAGELRGPPLQLDTGQLVKIAKRGGVTAVEAVHASRN 174
                *  .**.:.:* **  **:***.**  * ..** *.:.*:..:*: *  .*::*::  *.

RSc1815         KLTRAPLH 354
hpx17           KLTRAPLH 392
RScCAQ18687     KLTRAPLG 313
PthXo1          ALTGAPLN 182
                 ** ***
```

Figure S3. Alignment of N terminal sequences of Ralstonia TAL-like effectors (RTLs) and PthXo1. RTL sequences are those in GenBank with complete N-terminal and central repeat region sequences, RSc1815 (Genbank ID CAD15517.1) from *R. solanacearum* strain GMI1000, Hpx17 (Genbank ID AB178011.1) from strain RS1085, and RscCAQ18687 (Genbank ID CAQ18687.1) from strain MolK2. PthXo1 (Genbank ID ACD58243.1) is from *Xanthomonas oryzae* strain PXO99^{A}. Residues corresponding approximately to the -1st repeat are highlighted in light grey. Residues corresponding approximately to the 0th repeat are highlighted in darker grey. W232 and aligned arginine residues are shown in large bold type. Multiple sequence alignment was created with ClustalW [1].

1. Larkin MA, Blackshields G, Brown NP, Chenna R, McGettigan PA, et al. (2007) Clustal W and Clustal X version 2.0. Bioinformatics 23: 2947-2948.
