# Supplementary material for: TAL Effector Specificity for base 0 of the DNA Target Is Altered in a Complex, Effector- and Assay-Dependent Manner by Substitutions for the Tryptophan in Cryptic Repeat –1
Source: PLoS One. 2013 Dec 3;8(12):e82120. doi: 10.1371/journal.pone.0082120 (PMC3849474; doi:10.1371/journal.pone.0082120)
Supplement: Figure S4 — Secondary structure predictions for PthXo1 and Rsc1815 N-terminal regions. Left, PthXo1 (GenBank accession ACD58243.1) from Xanthomonas oryzae strain PXO99A. Right, RSc1815 (GenBank accession CAD15517.1) from Ralstonia solanacearum strain GMI1000. Secondary structures were predicted using Psipred [46]. (PDF) [file pone.0082120.s005.pdf]

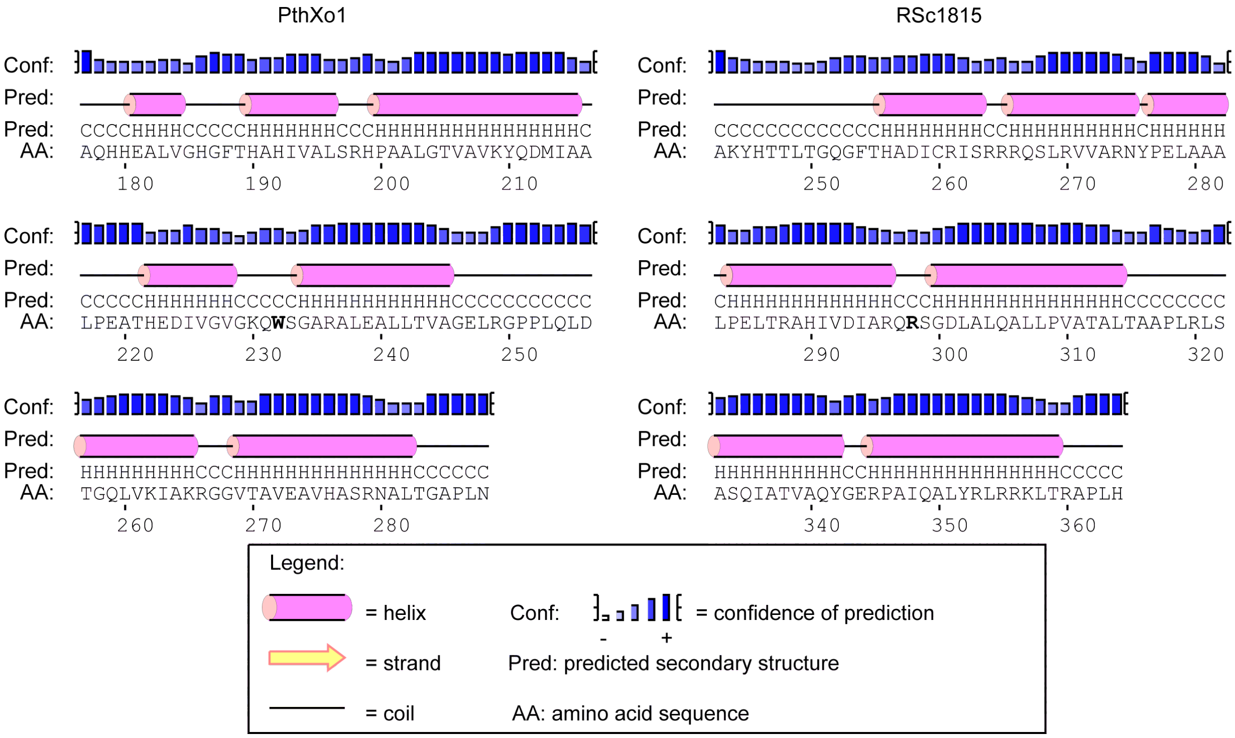

Figure S4. Secondary structure predictions for PthXo1 and Rsc1815 N-terminal regions. Left, PthXo1 (GenBank accession ACD58243.1) from *Xanthomonas oryzae* strain PXO99[A]. Right, RSc1815 (GenBank accession CAD15517.1) from *Ralstonia solanacearum* strain GMI1000. Secondary structure predictions were generated using Psipred [1]. W232 in PthXo1 and R298 in RSc1815 are in bold type.

1. Buchan DWA, Ward SM, Lobley AE, Nugent TCO, Bryson K, et al. (2010) Protein annotation and modelling servers at University College London. Nucleic Acids Research 38: W563-W568.
