# Supplementary material for: TAL Effector Specificity for base 0 of the DNA Target Is Altered in a Complex, Effector- and Assay-Dependent Manner by Substitutions for the Tryptophan in Cryptic Repeat –1
Source: PLoS One. 2013 Dec 3;8(12):e82120. doi: 10.1371/journal.pone.0082120 (PMC3849474; doi:10.1371/journal.pone.0082120)
Supplement: Table S1 — Oligonucleotides used for pAH236 W232 substitutions. (PDF) [file pone.0082120.s007.pdf]

Table S1. Oligonucleotides used for PthXo1 tryptophan 232 substitutions.

Mutation	Primer	Sequence[1]
All[2]	P885	5'-GCGTCGGCAAACAGGCGTCCGGCGCACGC-3'
W232A	P886	5'-GCGTGCGCCGGACGCCTGTTTGCCGACGC-3'
W232F	P919	5’-CGTGAGCAAGGCCTCCAGGGCTCGAGCGCCGGAAAACTGTTTGCCG-3’
W232L	P920	5’-CGTGAGCAAGGCCTCCAGGGCTCGAGCGCCGGATAACTGTTTGCCG-3’
W232I	P921	5’-CGTGAGCAAGGCCTCCAGGGCTCGAGCGCCGGAAATCTGTTTGCCG-3’
W232M	P922	5’-CGTGAGCAAGGCCTCCAGGGCTCGAGCGCCGGACATCTGTTTGCCG-3’
W232V	P923	5’-CGTGAGCAAGGCCTCCAGGGCTCGAGCGCCGGAGACCTGTTTGCCG-3’
W232S	P924	5’-CGTGAGCAAGGCCTCCAGGGCTCGAGCGCCGGAAGACTGTTTGCCG-3’
W232P	P925	5’-CGTGAGCAAGGCCTCCAGGGCTCGAGCGCCGGATGGCTGTTTGCCG-3’
W232T	P926	5’-CGTGAGCAAGGCCTCCAGGGCTCGAGCGCCGGATGTCTGTTTGCCG-3’
W232Y	P927	5’-CGTGAGCAAGGCCTCCAGGGCTCGAGCGCCGGAATACTGTTTGCCG-3’
W232H	P928	5’-CGTGAGCAAGGCCTCCAGGGCTCGAGCGCCGGAATGCTGTTTGCCG-3’
W232Q	P929	5’-CGTGAGCAAGGCCTCCAGGGCTCGAGCGCCGGATTGCTGTTTGCCG-3’
W232N	P930	5’-CGTGAGCAAGGCCTCCAGGGCTCGAGCGCCGGAATTCTGTTTGCCG-3’
W232K	P931	5’-CGTGAGCAAGGCCTCCAGGGCTCGAGCGCCGGATTTCTGTTTGCCG-3’
W232D	P932	5’-CGTGAGCAAGGCCTCCAGGGCTCGAGCGCCGGAATCCTGTTTGCCG-3’
W232E	P933	5’-CGTGAGCAAGGCCTCCAGGGCTCGAGCGCCGGATTCCTGTTTGCCG-3’
W232C	P934	5’-CGTGAGCAAGGCCTCCAGGGCTCGAGCGCCGGAACACTGTTTGCCG-3’
W232R	P935	5’-CGTGAGCAAGGCCTCCAGGGCTCGAGCGCCGGATCGCTGTTTGCCG-3’
W232G	P936	5’-CGTGAGCAAGGCCTCCAGGGCTCGAGCGCCGGATCCCTGTTTGCCG-3’

[1] Codons introducing W232 substitutions are highlighted in grey. The silent *Xho*1 site is underlined.
[2] Used in all reactions along with one of the primers listed below in the table to produce the desired mutation.
