# Supplementary material for: TAL Effector Specificity for base 0 of the DNA Target Is Altered in a Complex, Effector- and Assay-Dependent Manner by Substitutions for the Tryptophan in Cryptic Repeat –1
Source: PLoS One. 2013 Dec 3;8(12):e82120. doi: 10.1371/journal.pone.0082120 (PMC3849474; doi:10.1371/journal.pone.0082120)
Supplement: Table S2 — Oligonucleotides used for RTL-PthXo1 R298 substitutions. (PDF) [file pone.0082120.s008.pdf]

Table S2. Oligonucleotides used for RTL-PthXo1 R298 substitutions

Mutation	Primer	Sequence[1]
All[2]	PI178	5′-TTGCATGTAAATAGGAGGTGCACCATGAGAATAGGCAAATCAAG-3′
R232N	PI148	5′-CGGGTAGCAGCGCTTGCAGCGCCAGGTCACCCGAATTCTGCCGAGC-3′
R232P	PI147	5′-CGGGTAGCAGCGCTTGCAGCGCCAGGTCACCCGATGGCTGC-3′
R232Q	PI146	5′-CGGGTAGCAGCGCTTGCAGCGCCAGGTCACCCGATTGCTGC-3′
R232T	PI145	5′-CGGGTAGCAGCGCTTGCAGCGCCAGGTCACCCGATGTCTGC-3′
R232W	PI144	5′-CGGGTAGCAGCGCTTGCAGCGCCAGGTCACCCGACCACTGC-3′

[1] Codons introducing W232 substitutions are highlighted in grey
[2] Used in all reactions along with one of the primers listed below in the table to produce the desired mutation.
