# Supplementary material for: TAL Effector Specificity for base 0 of the DNA Target Is Altered in a Complex, Effector- and Assay-Dependent Manner by Substitutions for the Tryptophan in Cryptic Repeat –1
Source: PLoS One. 2013 Dec 3;8(12):e82120. doi: 10.1371/journal.pone.0082120 (PMC3849474; doi:10.1371/journal.pone.0082120)
Supplement: Table S3 — Oligonucleotides used for GUS reporter constructs. (PDF) [file pone.0082120.s009.pdf]

Table S3. Oligonucleotides used for construction of GUS reporter constructs.

EBE	Oligo 1	Oligo 2
EBE_PthXo1-T	5'-CGCGTGCATCTCCCCCTACTGTACACCAC-3'	5'-CGCGGTGGTGTACAGTAGGGGGAGATGCA-3'
EBE_PthXo1-A	5'-CGCGAGCATCTCCCCCTACTGTACACCAC-3'	5'-CGCGGTGGTGTACAGTAGGGGGAGATGCT-3'
EBE_PthXo1-C	5'-CGCGCGCATCTCCCCCTACTGTACACCAC-3'	5'-CGCGGTGGTGTACAGTAGGGGGAGATGCG-3'
EBE_PthXo1-G	5'-CGCGGGCATCTCCCCCTACTGTACACCAC-3'	5'-CGCGGTGGTGTACAGTAGGGGGAGATGCC-3′
EBE_868-T	5′-CGCGTACGTTAATGGAAGCT-3′	5′-CGCGAGCTTCCATTAACGTA-3′
EBE_868-A	5′-CGCGAACGTTAATGGAAGCT-3′	5′-CGCGAGCTTCCATTAACGTT-3′
EBE_868-C	5′-CGCGCACGTTAATGGAAGCT-3′	5′-CGCGAGCTTCCATTAACGTG-3′
EBE_868-G	5′-CGCGGACGTTAATGGAAGCT-3′	5′-CGCGAGCTTCCATTAACGTC-3′
