# Supplementary material for: TAL Effector Specificity for base 0 of the DNA Target Is Altered in a Complex, Effector- and Assay-Dependent Manner by Substitutions for the Tryptophan in Cryptic Repeat –1
Source: PLoS One. 2013 Dec 3;8(12):e82120. doi: 10.1371/journal.pone.0082120 (PMC3849474; doi:10.1371/journal.pone.0082120)
Supplement: Table S4 — Oligonucleotides used for W232 substitutions in TALEN yeast expression vectors. (PDF) [file pone.0082120.s010.pdf]

Table S4. Primers used for W232 substitutions in TALEN yeast expression vectors.

Change	Vector Backbone	Sequence[1]
All[2]	pZHY500 and pZHY501	5'-GGACGCAAGTGGTTGGTCTAGAATGGTGG-3'
W232N	pZHY500 and pZHY501	5'-CCTCCAGGGCGCGTGCGCCGGAATTCTGTTTGCCGACGCC-3'
W232P	pZHY500 and pZHY501	5'-CCTCCAGGGCGCGTGCGCCGGATGGCTGTTTGCCGACGCC-3'
W232Q	pZHY500 and pZHY501	5'-CCTCCAGGGCGCGTGCGCCGGATTGCTGTTTGCCGACGCC-3'
W232R	pZHY500 and pZHY501	5'-CCTCCAGGGCGCGTGCGCCGGATCGCTGTTTGCCGACGCC-3'
W232T	pZHY500 and pZHY501	5'-CCTCCAGGGCGCGTGCGCCGGATGTCTGTTTGCCGACGCC-3'

[1] Codons introducing W232 substitutions are highlighted in grey

[2] Used in all reactions along with one of the primers listed below in the table to produce the desired mutation.
