# Supplementary material for: TAL Effector Specificity for base 0 of the DNA Target Is Altered in a Complex, Effector- and Assay-Dependent Manner by Substitutions for the Tryptophan in Cryptic Repeat –1
Source: PLoS One. 2013 Dec 3;8(12):e82120. doi: 10.1371/journal.pone.0082120 (PMC3849474; doi:10.1371/journal.pone.0082120)
Supplement: Table S5 — Differences in size of effects on GUS activity between W232 substitutions co-delivered with targets with 0th A, C, G, or T in position 0 and the corresponding negative control. (PDF) [file pone.0082120.s011.pdf]

Table S5. Differences in size of effects on GUS activity between W232 substitutions co-delivered with targets with 0th A, C, G, or T in position 0 and the corresponding negative control.

Treatment A	Treatment B	Estimate[1]	SE[2]	Z statistic[3]	*p*-value[4]	Normalized Estimate[5]	Normalized SE[6]
**EBE_PthXo1-A + PthXo1**	EBE_PthXo1-T	**38.882**	**19.706**	**1.973**	**0.049**	**0.786**	**0.399**
**EBE_PthXo1-C + PthXo1**	EBE_PthXo1-T	**23.264**	19.706	1.181	0.238	0.471	0.399
**EBE_PthXo1-G + PthXo1**	EBE_PthXo1-T	**29.419**	19.706	1.493	0.136	0.595	0.399
**EBE_PthXo1-T + PthXo1**	EBE_PthXo1-T	**49.442**	14.785	3.344	0.001	1.000	0.299
**EBE_PthXo1-A + PthXo1 W232N**	EBE_PthXo1-T	**16.453**	22.586	0.729	0.466	0.333	0.457
**EBE_PthXo1-C + PthXo1 W232N**	EBE_PthXo1-T	**14.265**	22.515	0.634	0.526	0.289	0.455
**EBE_PthXo1-G + PthXo1 W232N**	EBE_PthXo1-T	**47.218**	22.515	2.097	0.036	0.955	0.455
**EBE_PthXo1-T + PthXo1 W232N**	EBE_PthXo1-T	**22.560**	22.971	0.982	0.326	0.456	0.465
**EBE_PthXo1-A + PthXo1 W232P**	EBE_PthXo1-T	**43.202**	19.027	2.271	0.023	0.874	0.385
**EBE_PthXo1-C + PthXo1 W232P**	EBE_PthXo1-T	**54.360**	19.027	2.857	0.004	1.099	0.385
**EBE_PthXo1-G + PthXo1 W232P**	EBE_PthXo1-T	**52.621**	19.027	2.766	0.006	1.064	0.385
**EBE_PthXo1-T + PthXo1 W232P**	EBE_PthXo1-T	**17.011**	19.027	0.894	0.371	0.344	0.385
**EBE_PthXo1-A + PthXo1 W232Q**	EBE_PthXo1-T	**36.786**	22.335	1.647	0.100	0.744	0.452
**EBE_PthXo1-C + PthXo1 W232Q**	EBE_PthXo1-T	**39.191**	22.335	1.755	0.079	0.793	0.452
**EBE_PthXo1-G + PthXo1 W232Q**	EBE_PthXo1-T	**119.879**	22.335	5.367	0.000	2.425	0.452
**EBE_PthXo1-T + PthXo1 W232Q**	EBE_PthXo1-T	**25.844**	22.335	1.157	0.247	0.523	0.452

**EBE_PthXo1-A + PthXo1 W232R**	EBE_PthXo1-T	**97.637**	25.512	3.827	0.000	1.975	0.516
**EBE_PthXo1-C + PthXo1 W232R**	EBE_PthXo1-T	**69.991**	25.512	2.743	0.006	1.416	0.516
**EBE_PthXo1-G + PthXo1 W232R**	EBE_PthXo1-T	**31.247**	25.512	1.225	0.221	0.632	0.516
**EBE_PthXo1-T + PthXo1 W232R**	EBE_PthXo1-T	**30.069**	25.512	1.179	0.239	0.608	0.516
**EBE_PthXo1-A + PthXo1 W232T**	EBE_PthXo1-T	**13.479**	18.784	0.718	0.473	0.273	0.380
**EBE_PthXo1-C + PthXo1 W232T**	EBE_PthXo1-T	**8.702**	18.784	0.463	0.643	0.176	0.380
**EBE_PthXo1-G + PthXo1 W232T**	EBE_PthXo1-T	**20.214**	18.784	1.076	0.282	0.409	0.380
**EBE_PthXo1-T + PthXo1 W232T**	EBE_PthXo1-T	**2.353**	18.985	0.124	0.901	0.048	0.384
**EBE_868-A + TAL868**	EBE_868-T	**35.327**	26.217	1.348	0.178	0.475	0.352
**EBE_868-C + TAL868**	EBE_868-T	**1.498**	26.217	0.057	0.954	0.020	0.352
**EBE_868-G + TAL868**	EBE_868-T	**2.811**	27.677	0.102	0.919	0.038	0.372
**EBE_868-T + TAL868**	EBE_868-T	**74.393**	16.979	4.381	0.000	1.000	0.228
**EBE_868-A + TAL868 W232N**	EBE_868-T	**20.644**	30.929	0.668	0.505	0.278	0.416
**EBE_868-C + TAL868 W232N**	EBE_868-T	**0.966**	30.929	0.031	0.975	0.013	0.416
**EBE_868-G + TAL868 W232N**	EBE_868-T	**20.786**	30.929	0.672	0.502	0.279	0.416
**EBE_868-T + TAL868 W232N**	EBE_868-T	**38.070**	30.929	1.231	0.218	0.512	0.416
**EBE_868-A + TAL868 W232P**	EBE_868-T	**33.484**	26.049	1.285	0.199	0.450	0.350
**EBE_868-C + TAL868 W232P**	EBE_868-T	**27.502**	26.049	1.056	0.291	0.370	0.350
**EBE_868-G +**	EBE_868-T	**38.097**	26.718	1.426	0.154	0.512	0.359

**TAL868 W232P**							
**EBE_868-T + TAL868 W232P**	EBE_868-T	**26.038**	26.049	1.000	0.318	0.350	0.350
**EBE_868-A + TAL868 W232Q**	EBE_868-T	**14.261**	26.588	0.536	0.592	0.192	0.357
**EBE_868-C + TAL868 W232Q**	EBE_868-T	**-4.184**	26.588	-0.157	0.875	-0.056	0.357
**EBE_868-G + TAL868 W232Q**	EBE_868-T	**31.152**	26.588	1.172	0.241	0.419	0.357
**EBE_868-T + TAL868 W232Q**	EBE_868-T	**41.774**	26.588	1.571	0.116	0.562	0.357
**EBE_868-A + TAL868 W232R**	EBE_868-T	**58.196**	23.575	2.469	0.014	0.782	0.317
**EBE_868-C + TAL868 W232R**	EBE_868-T	**51.425**	23.575	2.181	0.029	0.691	0.317
**EBE_868-G + TAL868 W232R**	EBE_868-T	**65.564**	23.575	2.781	0.005	0.881	0.317
**EBE_868-T + TAL868 W232R**	EBE_868-T	**70.968**	23.575	3.010	0.003	0.954	0.317
**EBE_868-A + TAL868 W232T**	EBE_868-T	**32.014**	26.199	1.222	0.222	0.430	0.352
**EBE_868-C + TAL868 W232T**	EBE_868-T	**3.401**	26.199	0.130	0.897	0.046	0.352
**EBE_868-G + TAL868 W232T**	EBE_868-T	**8.832**	26.199	0.337	0.736	0.119	0.352
**EBE_868-T + TAL868 W232T**	EBE_868-T	**33.922**	26.199	1.295	0.195	0.456	0.352

[1] Estimated difference between the effect sizes of Treatment A and Treatment B on GUS activity. Estimate<0 indicates Treatment A has a smaller effect on GUS activity than Treatment B. Estimate>0 indicates that Treatment A has a larger effect on GUS activity than Treatment B.
[2] Standard Error for the estimated difference in effect sizes computed by the general linear hypothesis test.
[3] Z statistic For the general linear hypothesis test of the null hypothesis that the expected value of the effect size of Treatment A is equal to the expected value of the effect size of Treatment B.
[4] p-value for the general linear hypothesis test of the null hypothesis that the expected value of the effect of Treatment A is equal to the expected value of the effect size of Treatment B.
[5] Calculated by dividing the estimate by the estimate of the difference in the effect sizes of the corresponding wild type TAL effector on the target with T at position 0 and the target with no co-delivered TAL effector, reported in Figure 2.
[6] Calculated in the same way as the Normalized Estimate. Reported as the error bars in Figure 2.
