# Supplementary material for: TAL Effector Specificity for base 0 of the DNA Target Is Altered in a Complex, Effector- and Assay-Dependent Manner by Substitutions for the Tryptophan in Cryptic Repeat –1
Source: PLoS One. 2013 Dec 3;8(12):e82120. doi: 10.1371/journal.pone.0082120 (PMC3849474; doi:10.1371/journal.pone.0082120)
Supplement: Table S6 — Differences in size of effects on GUS activity between W232 substitution variants co-delivered with targets with 0th position A, C, G, or T, and the wild-type TAL effector co-delivered with the target with 0th position T. (PDF) [file pone.0082120.s012.pdf]

Table S6. Differences in size of effects on GUS activity between W232 substitution variants co-delivered with targets with 0th position A, C, G, or T, and the wild type TAL effector co-delivered with the target with 0th position T.

Treatment A	Treatment B	Estimate[1]	SE[2]	Z statistic[3]	*p*-value[4]
**EBE_868-A + TAL868**	EBE_868-T + TAL868	-39.0658	26.2165	-1.4901	0.1362
**EBE_868-C + TAL868**	EBE_868-T + TAL868	-72.8951	26.2165	-2.7805	0.0054
**EBE_868-G + TAL868**	EBE_868-T + TAL868	-71.5824	27.6766	-2.5864	0.0097
**EBE_868-A + TAL868 W232N**	EBE_868-T + TAL868	-53.7488	30.9293	-1.7378	0.0822
**EBE_868-C + TAL868 W232N**	EBE_868-T + TAL868	-73.4265	30.9293	-2.374	0.0176
**EBE_868-G + TAL868 W232N**	EBE_868-T + TAL868	-53.6073	30.9293	-1.7332	0.0831
**EBE_868-T + TAL868 W232N**	EBE_868-T + TAL868	-36.3226	30.9293	-1.1744	0.2402
**EBE_868-A + TAL868 W232P**	EBE_868-T + TAL868	-40.9093	26.0485	-1.5705	0.1163
**EBE_868-C + TAL868 W232P**	EBE_868-T + TAL868	-46.8908	26.0485	-1.8001	0.0718
**EBE_868-G + TAL868 W232P**	EBE_868-T + TAL868	-36.2964	26.718	-1.3585	0.1743
**EBE_868-T + TAL868 W232P**	EBE_868-T + TAL868	-48.3547	26.0485	-1.8563	0.0634
**EBE_868-A + TAL868 W232Q**	EBE_868-T + TAL868	-60.132	26.5882	-2.2616	0.0237
**EBE_868-C + TAL868 W232Q**	EBE_868-T + TAL868	-78.577	26.5882	-2.9553	0.0031
**EBE_868-G + TAL868 W232Q**	EBE_868-T + TAL868	-43.2408	26.5882	-1.6263	0.1039
**EBE_868-T + TAL868 W232Q**	EBE_868-T + TAL868	-32.6187	26.5882	-1.2268	0.2199

**EBE_868-A + TAL868 W232R**	EBE_868-T + TAL868	-16.1969	23.5754	-0.687	0.4921
**EBE_868-C + TAL868 W232R**	EBE_868-T + TAL868	-22.9675	23.5754	-0.9742	0.3299
**EBE_868-G + TAL868 W232R**	EBE_868-T + TAL868	-8.8294	23.5754	-0.3745	0.708
**EBE_868-T + TAL868 W232R**	EBE_868-T + TAL868	-3.4251	23.5754	-0.1453	0.8845
**EBE_868-A + TAL868 W232T**	EBE_868-T + TAL868	-42.3792	26.1993	-1.6176	0.1058
**EBE_868-C + TAL868 W232T**	EBE_868-T + TAL868	-70.992	26.1993	-2.7097	0.0067
**EBE_868-G + TAL868 W232T**	EBE_868-T + TAL868	-65.5607	26.1993	-2.5024	0.0123
**EBE_868-T + TAL868 W232T**	EBE_868-T + TAL868	-40.4707	26.1993	-1.5447	0.1224
**EBE_868-A**	EBE_868-T + TAL868	-124.1506	26.2165	-4.7356	0
**EBE_868-C**	EBE_868-T + TAL868	-122.3079	26.2165	-4.6653	0
**EBE_868-G**	EBE_868-T + TAL868	-124.1598	26.2165	-4.7359	0
**EBE_868-T**	EBE_868-T + TAL868	-74.3929	16.9793	-4.3814	0
**EBE_PthXo1-A + PthXo1**	EBE_PthXo1-T + PthXo1	-10.56	19.0896	-0.5532	0.5801
**EBE_PthXo1-C + PthXo1**	EBE_PthXo1-T + PthXo1	-26.1776	19.0896	-1.3713	0.1703
**EBE_PthXo1-G + PthXo1**	EBE_PthXo1-T + PthXo1	-20.0229	19.0896	-1.0489	0.2942
**EBE_PthXo1-A + PthXo1 W232N**	EBE_PthXo1-T + PthXo1	-32.9886	22.5038	-1.4659	0.1427
**EBE_PthXo1-C + PthXo1 W232N**	EBE_PthXo1-T + PthXo1	-35.1768	22.4303	-1.5683	0.1168
**EBE_PthXo1-G + PthXo1 W232N**	EBE_PthXo1-T + PthXo1	-2.2242	22.4303	-0.0992	0.921

**EBE_PthXo1-T + PthXo1 W232N**	EBE_PthXo1-T + PthXo1	-26.8823	22.8873	-1.1746	0.2402
**EBE_PthXo1-A + PthXo1 W232P**	EBE_PthXo1-T + PthXo1	-6.2397	18.8549	-0.3309	0.7407
**EBE_PthXo1-C + PthXo1 W232P**	EBE_PthXo1-T + PthXo1	4.9183	18.8549	0.2609	0.7942
**EBE_PthXo1-G + PthXo1 W232P**	EBE_PthXo1-T + PthXo1	3.1788	18.8549	0.1686	0.8661
**EBE_PthXo1-T + PthXo1 W232P**	EBE_PthXo1-T + PthXo1	-32.4305	18.8549	-1.72	0.0854
**EBE_PthXo1-A + PthXo1 W232Q**	EBE_PthXo1-T + PthXo1	-12.6554	22.2111	-0.5698	0.5688
**EBE_PthXo1-C + PthXo1 W232Q**	EBE_PthXo1-T + PthXo1	-10.2513	22.2111	-0.4615	0.6444
**EBE_PthXo1-G + PthXo1 W232Q**	EBE_PthXo1-T + PthXo1	70.4372	22.2111	3.1713	0.0015
**EBE_PthXo1-T + PthXo1 W232Q**	EBE_PthXo1-T + PthXo1	-23.5981	22.2111	-1.0624	0.288
**EBE_PthXo1-A + PthXo1 W232R**	EBE_PthXo1-T + PthXo1	48.1952	25.4413	1.8944	0.0582
**EBE_PthXo1-C + PthXo1 W232R**	EBE_PthXo1-T + PthXo1	20.5489	25.4413	0.8077	0.4193
**EBE_PthXo1-G + PthXo1 W232R**	EBE_PthXo1-T + PthXo1	-18.1949	25.4413	-0.7152	0.4745
**EBE_PthXo1-T + PthXo1 W232R**	EBE_PthXo1-T + PthXo1	-19.3732	25.4413	-0.7615	0.4464
**EBE_PthXo1-A + PthXo1 W232T**	EBE_PthXo1-T + PthXo1	-35.9634	18.6278	-1.9306	0.0535
**EBE_PthXo1-C + PthXo1 W232T**	EBE_PthXo1-T + PthXo1	-40.7395	18.6278	-2.187	0.0287
**EBE_PthXo1-G + PthXo1 W232T**	EBE_PthXo1-T + PthXo1	-29.2283	18.6278	-1.5691	0.1166
**EBE_PthXo1-T + PthXo1 W232T**	EBE_PthXo1-T + PthXo1	-47.0892	18.8299	-2.5008	0.0124

**EBE_PthXo1-A**	EBE_PthXo1-T + PthXo1	-79.4279	52.4067	-1.5156	0.1296
**EBE_PthXo1-C**	EBE_PthXo1-T + PthXo1	-80.0302	52.4067	-1.5271	0.1267
**EBE_PthXo1-G**	EBE_PthXo1-T + PthXo1	-63.9657	52.4067	-1.2206	0.2223
**EBE_PthXo1-T**	EBE_PthXo1-T + PthXo1	-49.4419	14.7853	-3.344	0.0008

[1] Estimated difference between the effect sizes of Treatment A and Treatment B on GUS activity. Estimate<0 indicates Treatment A has a smaller effect on GUS activity than Treatment B. Estimate>0 indicates that Treatment A has a larger effect on GUS activity than Treatment B.
[2] Standard Error for the estimated difference in effect sizes computed by the general linear hypothesis test.
[3] Z statistic For the general linear hypothesis test of the null hypothesis that the expected value of the effect size of Treatment A is equal to the expected value of the effect size of Treatment B.
[4] *p*-value for the general linear hypothesis test of the null hypothesis that the expected value of the effect size of Treatment A is equal to the expected value of the effect size of Treatment B.
