# Supplementary material for: TAL Effector Specificity for base 0 of the DNA Target Is Altered in a Complex, Effector- and Assay-Dependent Manner by Substitutions for the Tryptophan in Cryptic Repeat –1
Source: PLoS One. 2013 Dec 3;8(12):e82120. doi: 10.1371/journal.pone.0082120 (PMC3849474; doi:10.1371/journal.pone.0082120)
Supplement: Table S7 — Differences in size of effects on GUS activity between W232 substitution variants co-delivered with targets with 0th position A, C, or G, and the same TAL effector co-delivered with the target with 0th position T, used to determine specificity for the 0th position. (PDF) [file pone.0082120.s013.pdf]

Table S7. Differences in size of effects on GUS activity between W232 substitution variants co-delivered with targets with 0th position A, C, or G, and the same TAL effector co-delivered with the target with 0th position T, used to determine specificity for the 0th position.

Treatment A	Treatment B	Estimate[1]	SE[2]	Z-statistic[3]	*p*-value[4]
**EBE_868-A + TAL868**	EBE_868-T + TAL868	-39.0658	26.2165	-1.4901	0.1362
**EBE_868-C + TAL868**	EBE_868-T + TAL868	-72.8951	26.2165	-2.7805	0.0054
**EBE_868-G + TAL868**	EBE_868-T + TAL868	-71.5824	27.6766	-2.5864	0.0097
**EBE_868-A + TAL868 W232N**	EBE_868-T + TAL868 W232N	-17.4261	37.9669	-0.459	0.6462
**EBE_868-C + TAL868 W232N**	EBE_868-T + TAL868 W232N	-37.1038	37.9669	-0.9773	0.3284
**EBE_868-G + TAL868 W232N**	EBE_868-T + TAL868 W232N	-17.2847	37.9669	-0.4553	0.6489
**EBE_868-A + TAL868 W232P**	EBE_868-T + TAL868 W232P	7.4455	30.9998	0.2402	0.8102
**EBE_868-C + TAL868 W232P**	EBE_868-T + TAL868 W232P	1.4639	30.9998	0.0472	0.9623
**EBE_868-G + TAL868 W232P**	EBE_868-T + TAL868 W232P	12.0584	31.5832	0.3818	0.7026
**EBE_868-A + TAL868 W232Q**	EBE_868-T + TAL868 W232Q	-27.5133	30.9998	-0.8875	0.3748
**EBE_868-C + TAL868 W232Q**	EBE_868-T + TAL868 W232Q	-45.9583	30.9998	-1.4825	0.1382
**EBE_868-G + TAL868 W232Q**	EBE_868-T + TAL868 W232Q	-10.6222	30.9998	-0.3427	0.7319
**EBE_868-A + TAL868 W232R**	EBE_868-T + TAL868 W232R	-12.7718	26.8466	-0.4757	0.6343
**EBE_868-C + TAL868 W232R**	EBE_868-T + TAL868 W232R	-19.5424	26.8466	-0.7279	0.4667
**EBE_868-G +**	EBE_868-T +	-5.4043	26.8466	-0.2013	0.8405

**TAL868 W232R**	TAL868 W232R				
**EBE_868-A + TAL868 W232T**	EBE_868-T + TAL868 W232T	-1.9085	30.9998	-0.0616	0.9509
**EBE_868-C + TAL868 W232T**	EBE_868-T + TAL868 W232T	-30.5213	30.9998	-0.9846	0.3248
**EBE_868-G + TAL868 W232T**	EBE_868-T + TAL868 W232T	-25.0901	30.9998	-0.8094	0.4183
**EBE_PthXo1-A + PthXo1**	EBE_PthXo1-T + PthXo1	-10.56	19.0896	-0.5532	0.5801
**EBE_PthXo1-C + PthXo1**	EBE_PthXo1-T + PthXo1	-26.1776	19.0896	-1.3713	0.1703
**EBE_PthXo1-G + PthXo1**	EBE_PthXo1-T + PthXo1	-20.0229	19.0896	-1.0489	0.2942
**EBE_PthXo1-A + PthXo1 W232N**	EBE_PthXo1-T + PthXo1 W232N	-6.1063	27.2481	-0.2241	0.8227
**EBE_PthXo1-C + PthXo1 W232N**	EBE_PthXo1-T + PthXo1 W232N	-8.2945	27.2235	-0.3047	0.7606
**EBE_PthXo1-G + PthXo1 W232N**	EBE_PthXo1-T + PthXo1 W232N	24.6582	27.2235	0.9058	0.3651
**EBE_PthXo1-A + PthXo1 W232P**	EBE_PthXo1-T + PthXo1 W232P	26.1908	21.5637	1.2146	0.2245
**EBE_PthXo1-C + PthXo1 W232P**	EBE_PthXo1-T + PthXo1 W232P	37.3488	21.5637	1.732	0.0833
**EBE_PthXo1-G + PthXo1 W232P**	EBE_PthXo1-T + PthXo1 W232P	35.6093	21.5637	1.6513	0.0987
**EBE_PthXo1-A + PthXo1 W232Q**	EBE_PthXo1-T + PthXo1 W232Q	10.9427	26.8466	0.4076	0.6836
**EBE_PthXo1-C + PthXo1 W232Q**	EBE_PthXo1-T + PthXo1 W232Q	13.3468	26.8466	0.4971	0.6191
**EBE_PthXo1-G + PthXo1 W232Q**	EBE_PthXo1-T + PthXo1 W232Q	94.0353	26.8466	3.5027	0.0005
**EBE_PthXo1-A + PthXo1 W232R**	EBE_PthXo1-T + PthXo1 W232R	67.5683	30.9998	2.1796	0.0293
**EBE_PthXo1-C +**	EBE_PthXo1-T +	39.9221	30.9998	1.2878	0.1978

**PthXo1 W232R**	PthXo1 W232R				
**EBE_PthXo1-G + PthXo1 W232R**	EBE_PthXo1-T + PthXo1 W232R	1.1782	30.9998	0.038	0.9697
**EBE_PthXo1-A + PthXo1 W232T**	EBE_PthXo1-T + PthXo1 W232T	11.1258	21.4063	0.5197	0.6032
**EBE_PthXo1-C + PthXo1 W232T**	EBE_PthXo1-T + PthXo1 W232T	6.3498	21.4063	0.2966	0.7667
**EBE_PthXo1-G + PthXo1 W232T**	EBE_PthXo1-T + PthXo1 W232T	17.8609	21.4063	0.8344	0.4041

[1] Estimated difference between the effect sizes of Treatment A and Treatment B on GUS activity. Estimate<0 indicates the Treatment A has a smaller effect on GUS activity than treatment B. Estimate>0 indicates that Treatment A has a larger effect on GUS activity than Treatment B.
[2] Standard Error for the estimated difference in effect sizes computed by the general linear hypothesis test.
[3] Z statistic For the general linear hypothesis test of the null hypothesis that the expected value of the effect size of Treatment A is equal to the expected value of the effect size of Treatment B.
[4] *p*-value for the general linear hypothesis test of the null hypothesis that the expected value of the effect size of Treatment A is equal to the expected value of the effect size of Treatment B.
