# Supplementary material for: TAL Effector Specificity for base 0 of the DNA Target Is Altered in a Complex, Effector- and Assay-Dependent Manner by Substitutions for the Tryptophan in Cryptic Repeat –1
Source: PLoS One. 2013 Dec 3;8(12):e82120. doi: 10.1371/journal.pone.0082120 (PMC3849474; doi:10.1371/journal.pone.0082120)
Supplement: Table S8 — Statistical significance of values shown in Figure 3, “Activity of TALENs with selected single amino acid substitutions for W232 on targets with A, C, G, or T at the 0th position.”. (PDF) [file pone.0082120.s014.pdf]

Table S8. Statistical significance of values shown in Figure 3, "Activity of TALENs with selected single amino acid substitutions for W232 on targets with A, C, G, or T at the 0th position."

TALEN/target	Number of samples (N)	Mean	SD	*p*-value cf. W232/T[a]	*p*-value cf. same/T[b]
PthXo1/EBE_PthXo1-A	11	0.52	0.06	0.0001	0.0001
PthXo1/EBE_PthXo1-C	11	0.63	0.07	0.0005	0.0005
PthXo1/EBE_PthXo1-G	11	0.28	0.09	0.0001	0.0001
PthXo1/EBE_PthXo1-T	10	1.00	0.28	1	1
PthXo1(W232N)/EBE_PthXo1-A	10	0.30	0.05	0.0001	0.0001
PthXo1(W232N)/EBE_PthXo1-C	10	0.22	0.02	0.0001	0.0001
PthXo1(W232N)/EBE_PthXo1-G	10	0.28	0.10	0.0001	0.0001
PthXo1(W232N)/EBE_PthXo1-T	9	0.71	0.13	0.01	1
PthXo1(W232P)/EBE_PthXo1-A	10	0.40	0.04	0.0001	0.0001
PthXo1(W232P)/EBE_PthXo1-C	10	0.35	0.05	0.0001	0.0001
PthXo1(W232P)/EBE_PthXo1-G	10	0.40	0.06	0.0001	0.0001
PthXo1(W232P)/EBE_PthXo1-T	9	0.76	0.06	0.0235	1
PthXo1(W232Q)/EBE_PthXo1-A	11	0.25	0.04	0.0001	0.0001
PthXo1(W232Q)/EBE_PthXo1-C	11	0.15	0.02	0.0001	0.0001
PthXo1(W232Q)/EBE_PthXo1-G	12	0.21	0.10	0.0001	0.0001
PthXo1(W232Q)/EBE_PthXo1-T	11	0.60	0.07	0.0002	1
PthXo1(W232R)/EBE_PthXo1-A	11	0.45	0.08	0.0001	0.0001
PthXo1(W232R)/EBE_PthXo1-C	11	0.28	0.04	0.0001	0.0001
PthXo1(W232R)/EBE_PthXo1-G	11	0.52	0.14	0.0001	0.0001
PthXo1(W232R)/EBE_PthXo1-T	10	0.81	0.08	0.0513	1
PthXo1(W232T)/EBE_PthXo1-A	12	0.36	0.04	0.0001	0.0005

PthXo1(W232T)/EBE_PthXo1-C	12	0.29	0.05	0.0001	0.0002
PthXo1(W232T)/EBE_PthXo1-G	12	0.29	0.07	0.0001	0.0002
PthXo1(W232T)/EBE_PthXo1-T	11	1.02	0.56	0.9265	1
TAL868/EBE_TAL868-A	15	0.86	0.47	0.3332	0.3332
TAL868/EBE_TAL868-C	15	0.75	0.50	0.1079	0.1079
TAL868/EBE_TAL868-G	15	0.52	0.48	0.0029	0.0029
TAL868/EBE_TAL868-T	15	1.00	0.31	1	1
TAL868(W232N)/EBE_TAL868-A	8	0.53	0.13	0.0007	0.0053
TAL868(W232N)/EBE_TAL868-C	8	0.87	0.17	0.3029	0.7013
TAL868(W232N)/EBE_TAL868-G	8	0.56	0.31	0.0038	0.0571
TAL868(W232N)/EBE_TAL868-T	8	0.83	0.22	0.2006	1
TAL868(W232P)/EBE_TAL868-A	7	0.38	0.29	0.0003	0.8081
TAL868(W232P)/EBE_TAL868-C	7	0.43	0.25	0.0005	0.9499
TAL868(W232P)/EBE_TAL868-G	7	0.37	0.24	0.0001	0.7159
TAL868(W232P)/EBE_TAL868-T	7	0.42	0.29	0.0005	1
TAL868(W232Q)/EBE_TAL868-A	8	0.60	0.13	0.0024	0.0003
TAL868(W232Q)/EBE_TAL868-C	8	0.99	0.19	0.9538	0.5088
TAL868(W232Q)/EBE_TAL868-G	8	0.86	0.19	0.2679	0.4008
TAL868(W232Q)/EBE_TAL868-T	8	0.94	0.15	0.5918	1
TAL868(W232R)/EBE_TAL868-A	8	0.45	0.14	0.0001	0.5350
TAL868(W232R)/EBE_TAL868-C	8	0.64	0.12	0.0055	0.0372
TAL868(W232R)/EBE_TAL868-G	8	0.57	0.09	0.0011	0.2123
TAL868(W232R)/EBE_TAL868-T	8	0.49	0.14	0.0003	1
TAL868(W232T)/EBE_TAL868-A	8	0.51	0.13	0.0004	0.0057
TAL868(W232T)/EBE_TAL868-C	8	1.07	0.21	0.5966	0.0125

TAL868(W232T)/EBE_TAL868-G	8	0.71	0.14	0.0234	0.4463
TAL868(W232T)/EBE_TAL868-T	8	0.78	0.19	0.0844	1

[a] *p*-value for the null hypothesis that the mean is equal to that of the unsubstituted TALEN on its EBE preceded by T, by two-tailed, unpaired *t*-test.
[b] *p*-value for the null hypothesis that the mean is equal to that of the same TALEN on its EBE preceded by T, by two-tailed, unpaired *t*-test.
