# Supplementary material for: TAL Effector Specificity for base 0 of the DNA Target Is Altered in a Complex, Effector- and Assay-Dependent Manner by Substitutions for the Tryptophan in Cryptic Repeat –1
Source: PLoS One. 2013 Dec 3;8(12):e82120. doi: 10.1371/journal.pone.0082120 (PMC3849474; doi:10.1371/journal.pone.0082120)
Supplement: Table S9 — Statistical significance of values shown in Figure 5, “Activity of chimeric TAL effector RTL-PthXo1 with single amino acid substitutions for R298 on targets with A, C, G, or T at the 0th position.”. (PDF) [file pone.0082120.s015.pdf]

Table S9. Statistical significance of values shown in Figure 5, "Activity of chimeric TAL effector RTL-PthXo1 with single amino acid substitutions for R298 on targets with A, C, G, or T at the 0th position."

Protein/target	Number of samples (N)	Mean	SD	*p*-value cf. None/T[a]	*p*-value cf. PthXo1/T[b]	*p*-value cf. same/T[c]
None/EBE_PthXo1-T	3	0.0223	0.0053	1.0000	0.0085	1.0000
PthXo1/EBE_PthXo1-T	3	1.0000	0.1579	0.0085	1.0000	1.0000
RTL-PthXo1/EBE_PthXo1-A	3	0.0266	0.0033	0.3091	0.0086	0.0210
RTL-PthXo1/EBE_PthXo1-C	3	0.0544	0.0055	0.0019	0.0091	0.2081
RTL-PthXo1/EBE_PthXo1-G	3	0.1290	0.0263	0.0166	0.0093	0.0379
RTL-PthXo1/EBE_PthXo1-T	3	0.0664	0.0117	0.0115	0.0091	1.0000
RTL-PthXo1(R298W)/EBE_PthXo1-A	3	0.0189	0.0040	0.4215	0.0085	0.2242
RTL-PthXo1(R298W)/EBE_PthXo1-C	3	0.0196	0.0043	0.5378	0.0085	0.3026
RTL-PthXo1(R298W)/EBE_PthXo1-G	3	0.0202	0.0016	0.5690	0.0085	0.2984
RTL-PthXo1(R298W)/EBE_PthXo1-T	3	0.0241	0.0049	0.6911	0.0086	1.0000
RTL-PthXo1(R298P)/EBE_PthXo1-A	3	0.0247	0.0042	0.0736	0.0277	0.0524
RTL-PthXo1(R298P)/EBE_PthXo1-C	3	0.0282	0.0025	0.0882	0.0280	0.1112
RTL-PthXo1(R298P)/EBE_PthXo1-G	3	0.0270	0.0027	0.0580	0.0279	0.0882
RTL-PthXo1(R298P)/EBE_PthXo1-T	3	0.0381	0.0067	0.2692	0.0285	1.0000
RTL-PthXo1(R298Q)/EBE_PthXo1-A	3	0.0278	0.0076	0.4150	0.0279	0.1619

RTL-PthXo1(R298Q)/EBE_PthXo1-C	3	0.0261	0.0015	0.0076	0.0278	0.0588
RTL-PthXo1(R298Q)/EBE_PthXo1-G	3	0.0242	0.0017	0.0041	0.0277	0.0402
RTL-PthXo1(R298Q)/EBE_PthXo1-T	3	0.0372	0.0053	0.2430	0.0284	1.0000
RTL-PthXo1(R298T)/EBE_PthXo1-A	3	0.0265	0.0035	0.4958	0.0247	0.8283
RTL-PthXo1(R298T)/EBE_PthXo1-C	3	0.0237	0.0028	0.1249	0.0245	0.4215
RTL-PthXo1(R298T)/EBE_PthXo1-G	3	0.0257	0.0015	0.2742	0.0246	0.6804
RTL-PthXo1(R298T)/EBE_PthXo1-T	3	0.0275	0.0065	0.8121	0.0247	1.0000
RTL-PthXo1(R298N)/EBE_PthXo1-A	3	0.0098	0.0052	0.0809	0.0125	0.0128
RTL-PthXo1(R298N)/EBE_PthXo1-C	3	0.0096	0.0015	0.0020	0.0125	0.0257
RTL-PthXo1(R298N)/EBE_PthXo1-G	3	0.0088	0.0021	0.0033	0.0125	0.0210
RTL-PthXo1(R298N)/EBE_PthXo1-T	3	0.0334	0.0073	0.0679	0.0130	1.0000

[a] *p*-value for the null hypothesis that the mean is equal to that of None/EBE_PthXo1-T, by two-tailed, unpaired *t*-test.
[b] *p*-value for the null hypothesis that the mean is equal to that of PthXo1/EBE_PthXo1-T, by two-tailed, unpaired *t*-test.
[c] *p*-value for the null hypothesis that the mean is equal to that of the same protein on EBE_PthXo1-T, by two-tailed, unpaired *t*-test.
